# Genomic profiling and spatial SEIR modeling of COVID-19 transmission in Western New York

**DOI:** 10.3389/fmicb.2024.1416580

**Published:** 2024-09-27

**Authors:** Jonathan E. Bard, Na Jiang, Jamaal Emerson, Madeleine Bartz, Natalie A. Lamb, Brandon J. Marzullo, Alyssa Pohlman, Amanda Boccolucci, Norma J. Nowak, Donald A. Yergeau, Andrew T. Crooks, Jennifer A. Surtees

**Affiliations:** ^1^Department of Biochemistry, Jacobs School of Medicine and Biomedical Sciences, State University of New York at Buffalo, Buffalo, NY, United States; ^2^Genomics and Bioinformatics Core, Jacobs School of Medicine and Biomedical Sciences, State University of New York at Buffalo, Buffalo, NY, United States; ^3^Department of Geography, State University of New York at Buffalo, Buffalo, NY, United States; ^4^Department of Microbiology and Immunology, Jacobs School of Medicine and Biomedical Sciences, State University of New York at Buffalo, Buffalo, NY, United States; ^5^National Renewable Energy Laboratory, Golden, CO, United States

**Keywords:** SARS-CoV-2, next-generation genome sequencing, spatial SEIR model, SARS-CoV-2 transmission dynamics, New York State

## Abstract

The COVID-19 pandemic has prompted an unprecedented global effort to understand and mitigate the spread of the SARS-CoV-2 virus. In this study, we present a comprehensive analysis of COVID-19 in Western New York (WNY), integrating individual patient-level genomic sequencing data with a spatially informed agent-based disease Susceptible-Exposed-Infectious-Recovered (SEIR) computational model. The integration of genomic and spatial data enables a multi-faceted exploration of the factors influencing the transmission patterns of COVID-19, including genetic variations in the viral genomes, population density, and movement dynamics in New York State (NYS). Our genomic analyses provide insights into the genetic heterogeneity of SARS-CoV-2 within a single lineage, at region-specific resolutions, while our population analyses provide models for SARS-CoV-2 lineage transmission. Together, our findings shed light on localized dynamics of the pandemic, revealing potential cross-county transmission networks. This interdisciplinary approach, bridging genomics and spatial modeling, contributes to a more comprehensive understanding of COVID-19 dynamics. The results of this study have implications for future public health strategies, including guiding targeted interventions and resource allocations to control the spread of similar viruses.

## 1 Introduction

The global impact of the COVID-19 pandemic has been profound, necessitating an unprecedented global response to understand, manage, and mitigate the spread of the SARS-CoV-2 virus (Vallée, 2023; Sawicka et al., 2022). The novel coronavirus has traversed borders and affected communities on a scale that demands comprehensive research and innovative strategies for public health management (Miyah et al., 2022). Amidst this global challenge, a critical aspect that emerged is the importance of understanding and addressing local transmission dynamics (Foraker et al., 2021). While the broader picture of the pandemic is crucial, the intricacies of how the virus spreads within specific localities are essential for effective public health interventions. Local transmission dynamics not only shape the trajectory of the pandemic but also influence the efficacy of control measures and resource allocation (Emanuel et al., 2020; Wurmb et al., 2020). For this analysis, we have focused on the Western region of New York State (WNY), which is characterized by both metropolitan and rural communities with varying population densities. We sought to unravel the unique regional factors influencing COVID-19 transmission within these communities.

To develop a comprehensive understanding of COVID-19 dynamics, we employed a layered approach that combines detailed genomic analysis of SARS-CoV-2 lineages with spatially informed SEIR models (Jiang et al., 2022). Our genomic sequencing analysis allowed us to investigate the diversity and evolution of SARS-CoV-2 lineages within NYS. Characterizing viral genetic variations at the individual patient level revealed significant heterogeneity of viral genomes at the sub-lineage level within and between geographic regions. Our SEIR models build on genomic and geographic data to provide a dynamic framework for simulating disease spread based on population movements and epidemiological parameters. In these models, agents (synthetic individuals) occupy Susceptible, Exposed, Infectious, and Recovered states (Kong et al., 2022; Shankar et al., 2021). For our model, we first established regional commuter dynamics using state-wide traffic data, followed by more granular census-tract estimations across different social networks (i.e., home, work, and school). Our spatially aware modeling strategy allowed us to simulate and analyze potential transmission patterns between distinct areas around WNY, accounting for local factors such as population density and commuter movement between neighboring areas (Wong et al., 2023).

Thus, the combination of SEIR models and genomic analysis not only enhances our ability to predict and understand the spread of COVID-19, but also provides a unique perspective on how viral genetic variations may contribute to regional differences in transmission dynamics. Our approach offers a robust framework for unraveling the intricate interplay between population-level movement dynamics and viral evolution in the context of the ongoing pandemic.

## 2 Materials and methods

### 2.1 SARS-CoV-2 patient sequencing data and regional analysis

SARS-CoV-2 viral genomes were accessed and downloaded from the GISAID database for 2020–2022, and filtered to New York, United States and Ontario, Canada (Bogner et al., 2006; Khare et al., 2021). The collection date, county, and lineages provided in the GISAID metadata text files were aggregated using the R programming language and plotted using the packages ggplot2 (Wickham, 2016), lubridate (Grolemund and Wickham, 2011), and tidyverse. For the complete GISAID dataset for the spatial analysis of Ontario and New York State, GISAID EPI_SET ID EPI_SET_231204fx 10.55876/gis8.231204fx. For the B.1.1.7 analysis, GISAID EPI_SET ID EPI_SET_231204bh 10.55876/gis8.231204bh. For the BA.2.12.1 analysis, the GISAID EPI_SET ID is EPI_SET_231204dh 10.55876/gis8.231204dh. County-level per 100,000 resident normalizations were based on the 2020 census data.

### 2.2 SARS-CoV-2 economic development region rank-correlation coefficients and UMAP reduction

GISAID metadata from 2020 to 2022 were downloaded and filtered for New York State and Ontario, Canada. Location information was post-processed to group by Economic Development Region (EDR). The metadata was then collapsed by Date Collection, EDR, and Summation for each Pango Lineage designation. Next, for each EDR, we calculated the relative abundance ranking for each lineage and correlated each EDR with all other EDRs. The resulting similarity matrix was next visualized in R. Uniform manifold approximation and projection (UMAP) analysis was performed using the R package UWOT on the sample counts for each lineage, for each EDR, grouped by year. The local neighbor’s parameter was set to n_neighbors = 2. The dimension reduction results were visualized using the R package ggplot2.

### 2.3 Genomic clustering and phylogenetic analysis using the Jaccard metric

Variant profiles for each viral genome were compared using the bedtools Jaccard function (Quinlan and Hall, 2010). The Jaccard statistic is a similarity coefficient defined as the intersection size divided by the size of the union of two sets (in this case, the variant profiles for each sample being compared). The resulting similarity matrix was input into the R pheatmap package for hierarchical clustering and annotated by the county of origin. For phylogenetic analysis, consensus genomes were aligned using the command line version of the MAFFT multiple sequencing alignment algorithm (Katoh et al., 2019). The resulting alignment was then input into the FastTree algorithm, inferring maximum-likelihood phylogeny using the jukes-cantor distance model of nucleotide evolution, generating a newick formatted phylogenetic tree (Price et al., 2010). The R packages TreeIO (Wang et al., 2020) and ggtree (Yu, 2020) were used for data visualization, with the Pango lineage metadata as data overlays (Rambaut et al., 2020). For BA.2.12.1 sublineage analysis, sample-to-sample distances were derived from the phylogenetic tree, and used as input into k-means clustering with k = 4.

### 2.4 NYS thruway datasets and traffic info

NYS Thruway data was accessed via the data.ny.gov browser (Authority, N. Y. S. T, 2020). Data records for 2019 thruway usage were accessed, and entries from the database that did not correspond to the main-line thruway were removed. Entrance and Exit points were aggregated over time, and the subsequent contact matrix was plotted in R following log normalized z-scoring.

### 2.5 Spatial susceptible-exposed-infections-recovered model

Using agent-based modeling (Crooks et al., 2019), we have built a spatial SEIR model at the individual population level for WNY to demonstrate how commuter activity could lead to the diffusion of SARS-CoV-2. To build the model, three steps were involved: first, the creation of a synthetic population and its corresponding social networks; second, building of an agent-based SEIR model using the synthetic population; and lastly, analysis of the simulation results. The model logic and full details of the model are provided along with the source code and data needed to replicate the results. It is available at https://figshare.com/articles/software/2_Agent-based_SEIR_model/24711945.

For the first step, the synthetic population and its social networks were created by following the method previously reported by Jiang et al. (2022) using census data, which are taken as input parameters to initialize the agents of the SEIR model. Specifically, we used datasets from the US Census for home and work locations and the U.S. Environmental Protection Agency (EPA) for school locations (U.S Census Bureau, 2022). Within the synthetic population, individuals are either children (i.e., ages <18) or adults (i.e., ages ≥18). Our model assumes children of school age go to their closest schools or daycares or stay at home with their parents, while adults commute to work or stay home. The work commute information for adults is consistent with the U.S. Census Bureau’s Longitudinal Employer-Household Dynamics (LEHD) Origin–Destination Employment Statistics (LODES) data. Then, we constructed social networks (i.e., home, work, and education). These were created based on the small-world network principle (Newman and Watts, 1999), where the synthetic individuals are connected based on living in the same household and working in the same workplace or attending the same daycare/education institute. Small-world networks are created for people whose workplace has more than 5 people, where the number 5 is chosen to indicate the size of the core social group with 5 people based on the work of Dunbar (1998), which still holds in current society (West et al., 2020; Tamarit et al., 2022). To mimic the core social group of 5 people, k = 4, which means one person can be connected to 4 people to make up a 5 people social group; p is set as 0.3, which indicates the probability of adding a new edge for each edge, to enable us to have a variation on edges, allowing for some agents to have more or fewer connections.

The rationale for these networks is that an individual might go to work, become exposed to COVID-19, and then go home and infect family members who in turn go to a school and infect students at school, propagating the viral infection through the network. It should be noted that the size of the social networks can be adjusted within the synthetic population code. Interested readers can use the provided code to explore the effects of network sizes.

After the synthetic population and its social networks were built, step two involved the creation of the agent-based model. Parameters related to the lineages (e.g., R0, incubation, and recovery period) are used for the initialization of heterogenous agents. Specifically, the model assumes a basic reproductive number (i.e., R0) as 3; while 7 to 14 days for the incubation period and 4 to 14 for the recovery period (Achaiah et al., 2020; Wu et al., 2022). Then, SEIR statuses are integrated into the agents to represent their health status. In this model, a time step represents 8 hours, where 1 day is divided into 3 time periods, which are characterized as being at home (i.e., either sleeping or getting up), at work (i.e., at work or educational site) and at home (i.e., back at home from work or educational site). The agents interact (i.e., spread the disease or get infected) through their social networks at each time step. In this model, we consider agents who have a work social network as commuters. When these agents are at work, they will only interact with agents in their work (or school) social network. While agents are at home, they only interact with members from the same household social network. If there is one commuter in the household, the rest of the members of the household have the potential to be infected by the commuter. We should note that all models are simplifications of reality, and, in this model, we chose not to model infections on public transport or outside of home and work for simplicity, but also due to the limited available data (e.g., lack of frequency of public transport, and its capacity). However, we feel that home and work dynamics are sufficient in this example model as these are where most people spend most of their time. We note that this model can be modified with additional input data. The model has been programmed to track the overall SEIR dynamics each day (i.e., every 3-time steps) and generates a dataset comprising infectious agents’ information every 10 days (i.e., 30-time steps) during the simulation, which is then analyzed in step 3 (as we will discuss in Section 4).

## 3 Results

### 3.1 Statewide variation of major SARS-CoV-2 lineages over time

**Figure 1:**
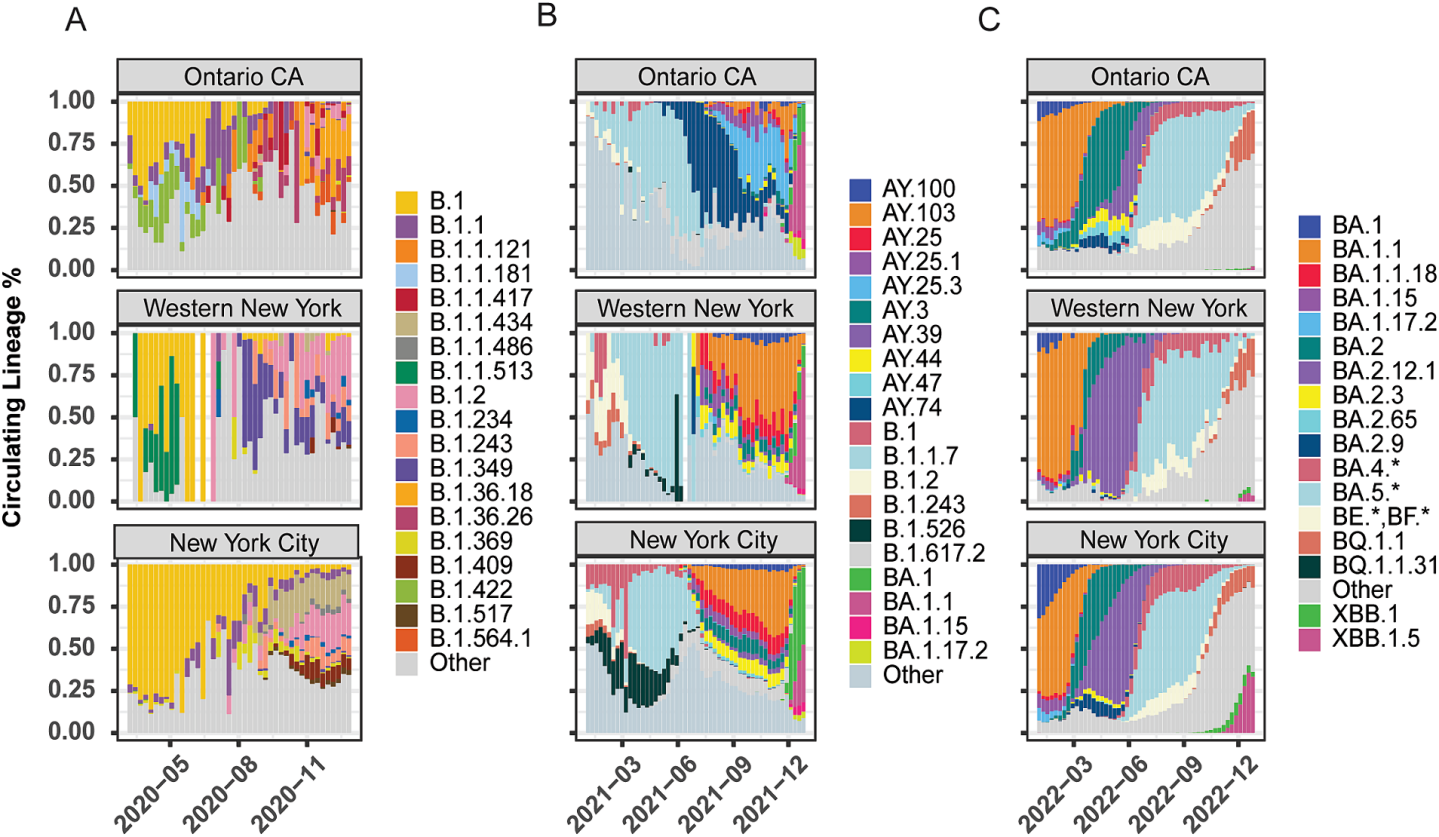
Lineage distribution of SARS-CoV-2 across geographic regions. Lineage distributions by percentage of total cases within region per week across Ontario, Canada, Western New York, and New York City. **(A)** 2020 proportions by week (March – December). **(B)** 2021 proportions by week (January–December). **(C)** 2022 proportions by week (January – December). The “Other” category represents the collection of lineages present with <1% of the total lineages and lacking discrete regional patterning.

Our goal was to establish whether there were regional differences in the SARS-CoV-2 lineages circulating across NYS. We analyzed the lineage distributions across 10 Economic Development Regions (EDR) in NYS and Ontario, Canada (Supplementary Figure 1). To assess broad differences at either end of NYS, we tracked reported caseloads by lineage and location in the WNY and New York City (NYC) areas over 2020, 2021, and 2022 (Figure 1). We also included the Canadian providence of Ontario (OCA) in our analysis due to the proximity and large commuter population between the southern portion of Ontario (Niagara Falls, Ontario and Fort Erie, Ontario to Buffalo, New York and Niagara Falls, New York; Authority, B. A. F. E. P. B, 2023). Data for the eight EDRs between WNY and NYC are shown in Supplementary Figures 2–4. For each week through the pandemic, we present a histogram representing the proportion of each lineage observed in a given region. Lineages selected for graphical display were chosen due to distinct regional patterns and for overall abundance across NYS. Lineages not reaching a proportion greater than ~1% were combined as “Other” and are shown in grey in the histograms (Figure 1; Supplementary Figures 2–4). The details of all lineages, including those not shown in the histograms, are reported in Supplementary Tables 2–4.

In 2020, we observed distinct lineages between Ontario and NYS (Figure 1A; Supplementary Figure 2). Early in 2020, all three regions (OCA, WNY, NYS) were high in the B.1 lineage (yellow), but very shortly after regionally specific lineage profiles began to emerge, likely driven by the strong lock-down measures of the early pandemic in combination with evolution of the virus. Early in the pandemic, lineages B.1.422 (light green) and B.1.1.181 (light blue) were uniquely detected in Ontario, along with B.1.1 (purple) which also had some presence in NYC. Later, Ontario saw increased levels of B.1.1, B.1.1.121(orange) and B.1.1.417 (dark red), while WNY showed increased levels of B.1.2 (pink), and uniquely elevated levels of B.1.1.513 (dark green). Conversely, New York City was dominated by B.1 through the summer, with some B.1.1 and B.1.2, but had little to no cases of either B1.1.181 or B.1.422. Toward August and September of 2020, Western New York saw increased levels of B.1.349 (indigo) compared to Ontario and NYC (Figure 1A). Conversely, lineages B.1.1.434 (tan), B.1.369 (chartreuse), and B.1.409 (maroon) were specific to New York City (with the Mid-Hudson region also predominantly B.1.1.434). Interestingly, the Capital Region shared elements of Western New York and New York City profiles (Supplementary Figure 2). These data thus highlight the distinct regional differences at opposite ends of NYS during 2020 (Figure 1A).

Late 2020 through early 2021 saw the introduction of major variants of concern to the U.S. Our data indicates that the introduction of Alpha (B.1.1.7; pale blue) replaced the regional differences across NYS (Supplementary Figure 3) and in Ontario, although the timing of its dominance varied by region. In Ontario, B.1.1.7 was subsequently replaced by Delta-based lineages B.1.617.2 (pale grey) and then AY.74 (navy) by June 2021, which did not see widespread transmission in NYS (Figure 1B; Supplementary Figure 3). In NYS, B.1.526 (Iota variant; black) saw strong regional effects, with very little transmission in WNY, Finger Lakes (FL), Central New York (CNY), and OCA, while seeing upwards of 25% in Mohawk Valley (MV), NYC, Capital Region (CR), Mid-Hudson (MH), and Long Island (LI; Figure 1B; Supplementary Figure 3). More heterogeneity is seen in NYS following the introduction of Delta and its various AY offshoots; AY.103 (orange) and AY.25 (magenta) were not present in OCA, but seen across NYS (Figure 1B; Supplementary Figure 2). Lastly, BA.1 (Omicron variant; green) appeared in late 2021. However, cases of this variant were primarily seen in NYC, MH, and LI, with Omicron variant BA.1.1 dominating in WNY and OCA (Figure 1B; Supplementary Figure 3). These results suggest that regionally specific lineages were circulating in NYS, and these regional differences were maintained throughout the pandemic, even after the introduction of primary variants of concern (Alpha and Delta) at the beginning of 2021. In 2022, the Omicron lineage dominated all surveyed regions, and the lineage distributions appear more similar across NYS and Ontario. Nonetheless, regional differences in the proportion and timing of distinct sub-lineages persisted. In OCA, a much higher proportion of BA.2 (dark teal) was observed and for longer than in NYS. BA.2.3 (yellow), BA.2.65 (light blue), BA.2.9 (navy) were observed in higher proportions in OCA than in NYS; BA.2.9 was not detected in WNY, Southern Tier (ST), FL, CNY, MH, and North Country (NC; Figure 1C; Supplementary Figure 4).

**Figure 2:**
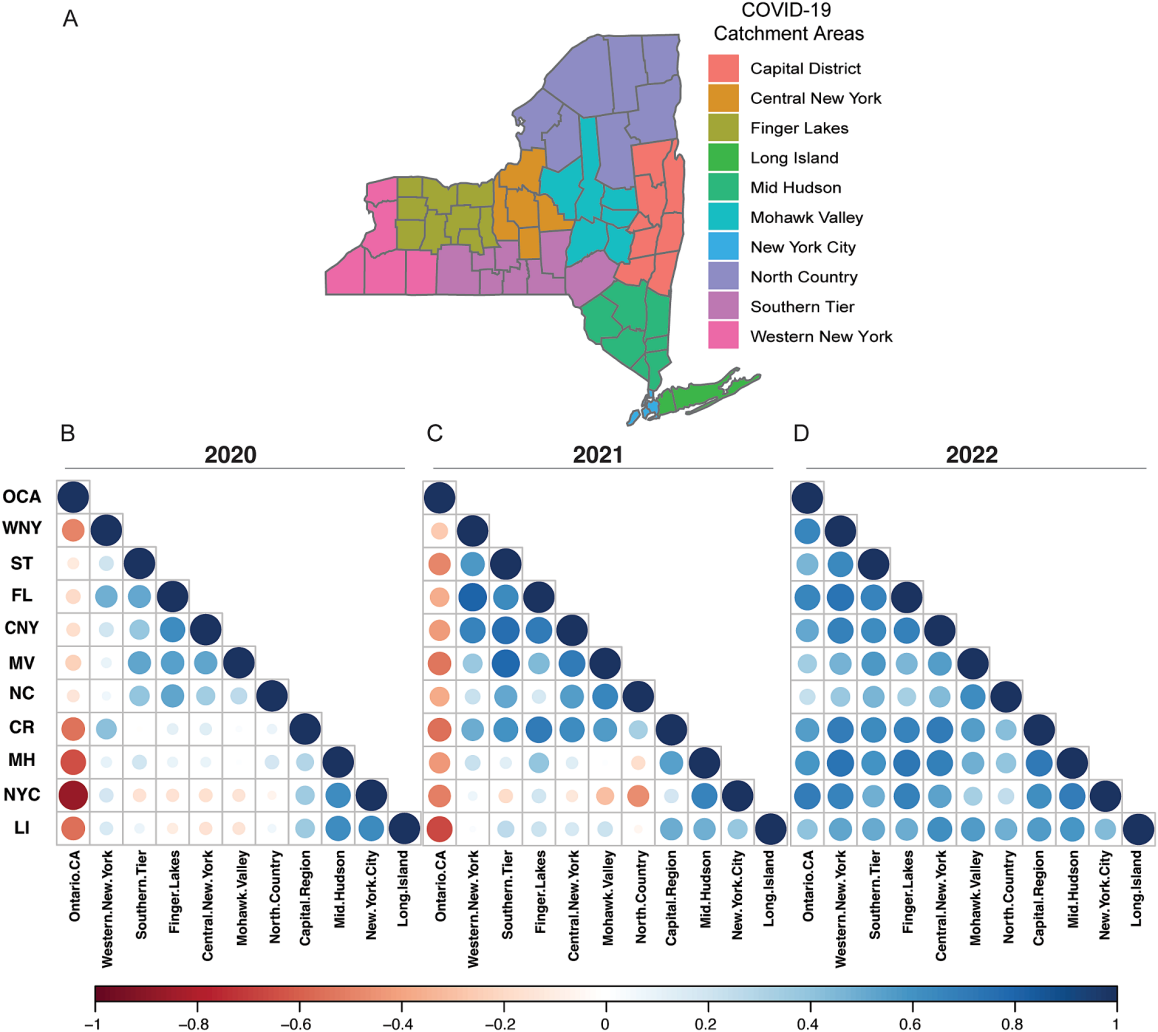
Geographic economic development regions and lineage correlations. **(A)** New York State county grouping into EDR regions. Not shown OCA. **(B)** EDR Rank-Correlation Coefficient matrix for 2020. **(C)** EDR Rank-Correlation Coefficient matrix for 2021. **(D)** EDR Rank-Correlation Coefficient matrix for 2022. All correlations using the Pearson correlation coefficient.

We analyzed lineage data from each EDR spanning 2020–2022 using rank-correlation coefficient analysis (Figure 2). For each EDR, we correlated the relative rankings to all other EDRs and OCA. In 2020, we observed a negative correlation (shades of red) between OCA and all NYS regions (Figure 2B). Conversely, there were higher correlations (shades of blue) between the geographically close regions within NYS, i.e., between MV, CNY, FL, and NC regions, and between downstate MH, NYC, and LI regions (Figure 2B). In 2021, following decreased lockdown restrictions, the correlation between lineages circulating increased between most NYS EDRs, though OCA was still displaying unique distributions (Figure 2C). Finally, by 2022 we saw a dramatic normalization of the lineages circulating across all EDRs, including OCA (Figure 2D). To further support our rank-correlation analysis, for each EDR, we analyzed lineage distributions using uniform manifold approximation and projection (UMAP) dimension-reduction (Supplementary Figure 5). Like our rank-correlation coefficient analysis, in 2020 we saw increased dispersion in the UMAP analysis (Supplementary Figure 5A), as compared to 2021 and 2022 (Supplementary Figures 5B,C). For both analyses, it is important to note that the number of viral genomes sequenced decreased rapidly in the latter half of 2022, resulting in sparse coverage for several EDRs including MV, NC, and LI which may explain slightly lower correlations to other EDRs.

### 3.2 Spatial and temporal modeling of SARS-CoV-2 nucleotide polymorphisms

**Figure 3:**
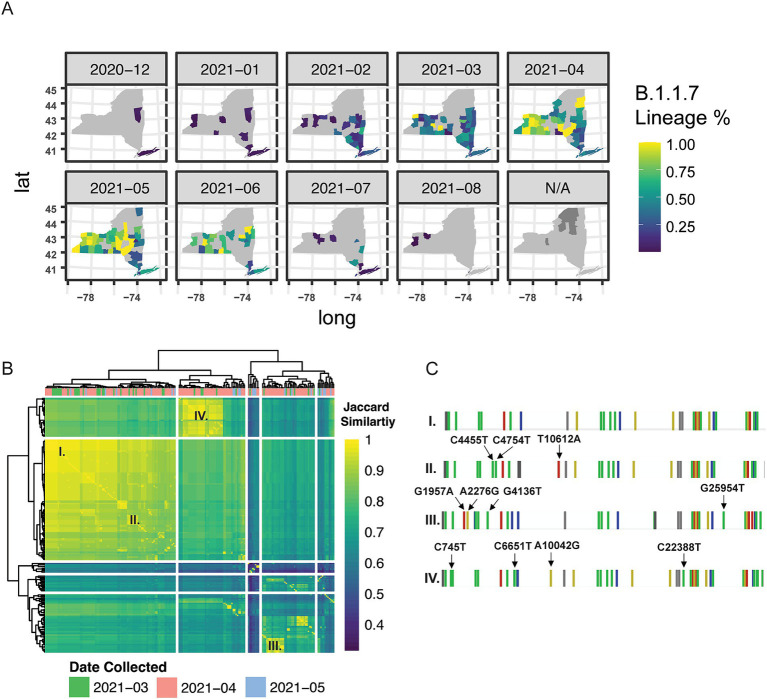
Genetic and spatial–temporal distribution of alpha B.1.1.7. **(A)** Geographic introduction and organization of B.1.1.7 lineage from December 2020 to August 2021, by percentage of SARS-CoV-2 circulating in each county per month. N/A represents counties with no B.1.1.7 cases sequenced. **(B)** Hierarchical clustering of Jaccard distance estimations between variant profiles of 200 Erie County B.1.1.7 samples from March–May 2021; cases were very low in June – August (9 sequences total were available) and therefore we excluded this time period. **(C)** Single base-pair mutational profiles for select B.1.1.7 samples within Erie County.

Based on our findings that there were strong regional relationships on the spread of SARS-CoV-2 lineages across New York State, we next sought to quantify whether we could detect nucleotide level differences in samples belonging to the same variant-of-concern lineage within a single-county in WNY. We first evaluated Alpha (B.1.1.7) in Erie County (WNY) as a proof-of-principle analysis. B.1.1.7 was first introduced in NYS in early December 2020, likely because of air travel between the United Kingdom and one of the New York City Airports (Alpert et al., 2021). Our temporal analysis of genomic lineages indicated that B.1.1.7 was also introduced in WNY with similar timing (Figure 1; Figure 3A). At the same time, spatiotemporal analysis of case numbers per county indicates that B.1.1.7 spread up the Hudson Valley and across NYS over a six-month period (Figure 3A). We posited that the B.1.1.7 detected in Erie County then was due to multiple introductions, which could be determined by distinct nucleotide polymorphisms within the genomes of sequenced viruses. To test this, we evaluated 200 B.1.1.7 samples collected in Erie County, New York, between March 2021 and May 2021, and assessed the similarities between viral genomes (Figure 3B). We found several distinct patterns of mutations present in B.1.1.7, lending support to our hypothesis of multiple introductions of B.1.1.7 in Erie County (Figure 3C).

**Figure 4:**
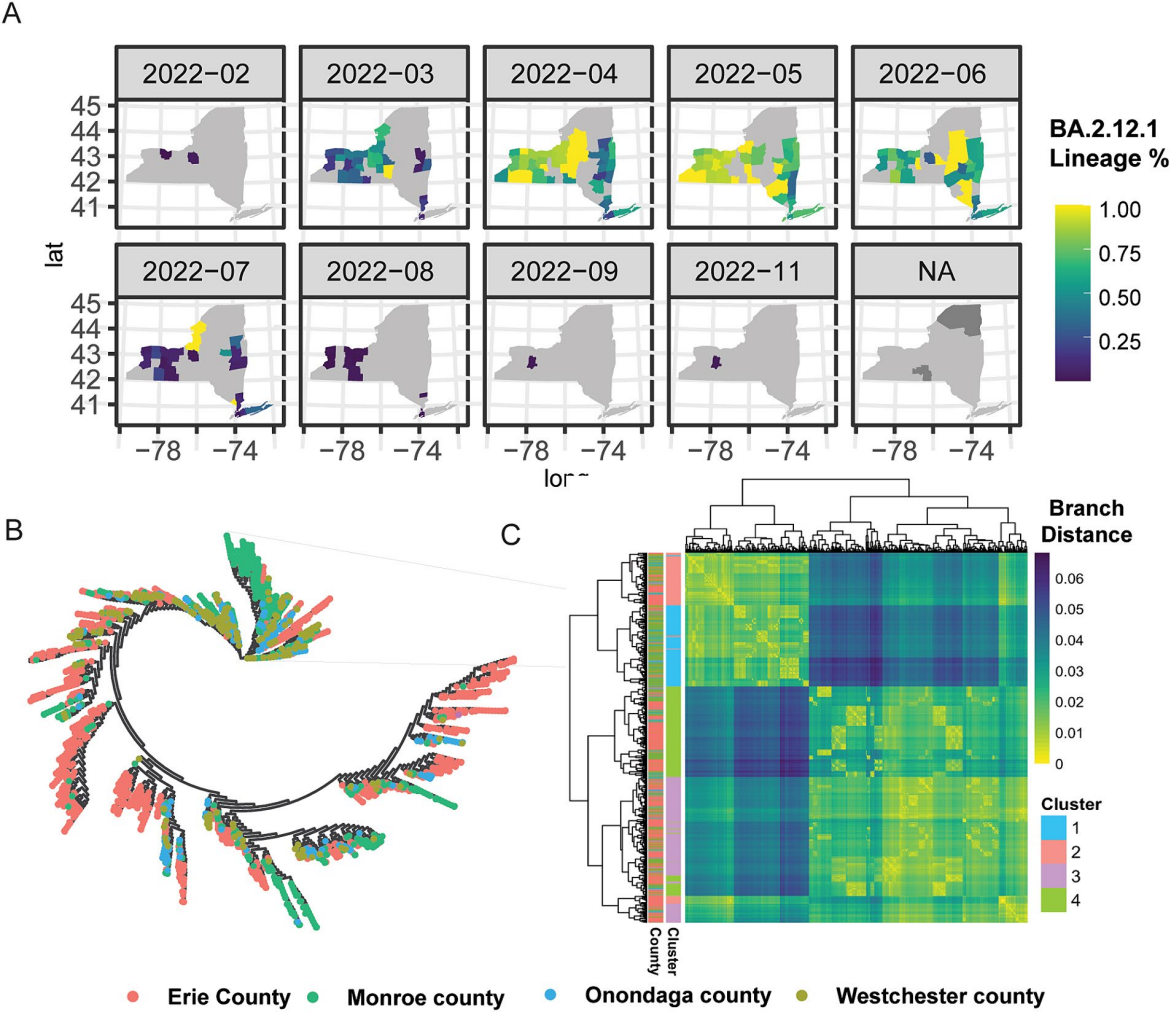
Phylogenetic and spatial–temporal distribution of omicron BA.2.12.1. **(A)** Geographic introduction and organization of BA.2.12.1 lineage from February 2022 to November 2022, by percentage of SARS-CoV-2 circulating in each county per month. N/A represents counties with no BA.2.12.1 cases sequenced. **(B)** Phylogenetic clustering of jukes-cantor distance estimations between consensus sequences of 2,737 samples. Lineages on the phylogenetic tree are color-coded by county; Erie County (pink), Monroe County (green), Onondaga County (blue), and Westchester County (chartreuse). **(C)** Hierarchical clustering of sample-to-sample distance estimation of 2,737 BA.2.12.1 lineages in four counties across NYS, with k-means clustering k = 4.

Encouraged by the analysis of B.1.1.7, we next theorized that there would exist larger mutational differences within a lineage between major metropolitan regions. We next selected Omicron BA.2.12.1 for in-depth analysis due to its high virulence and immune evasion potential, as well as robust sample counts across NYS (Figure 4A; Cao et al., 2022, Beheshti Namdar and Keikha, 2022). Unlike in the case of B.1.1.7, we saw earlier introduction of BA.2.12.1 to Monroe (FL EDR) and Onondaga (CNY EDR) Counties, where it quickly spread and became dominant in the Central and Western portion of NYS, in addition to the Capital Region, the Hudson Valley and into NYC and Long Island (Figure 4A). Intriguingly, phylogenetic analysis of 2,737 samples from Erie County (WNY), Monroe County (FL), Onondaga County (CNY), and Westchester County (MH) revealed distinct genomic groupings between the different geographically located regions (Figure 4B). Each branch on the phylogenetic tree is dominated by a single color, representing a distinct county. Of the samples profiled, each county demonstrates distinct genomic profiles for BA.2.12.1, further supporting the notion that tracking lineages at the nucleotide level reveals distinct region-specific alterations that are otherwise hidden by broad lineage designations (Figures 4B,C). To further elucidate the spatial–temporal distribution of these distinct lineages, samples were grouped using kmeans clustering (*k* = 4; Figure 4C), and each cluster distribution was plotted over time (Supplementary Figure 6). Cluster 1 was first detected in Monroe and Onondaga County, while C4 was first seen in Onondaga. Conversely, C2 and C3 appeared across several counties. These results, taken with our analysis of Erie County B.1.1.7, highlight nuanced differences of lineages circulating at the genetic level with regional patterning.

### 3.3 Establishing broad travel patterns using transit dataset

**Figure 5:**
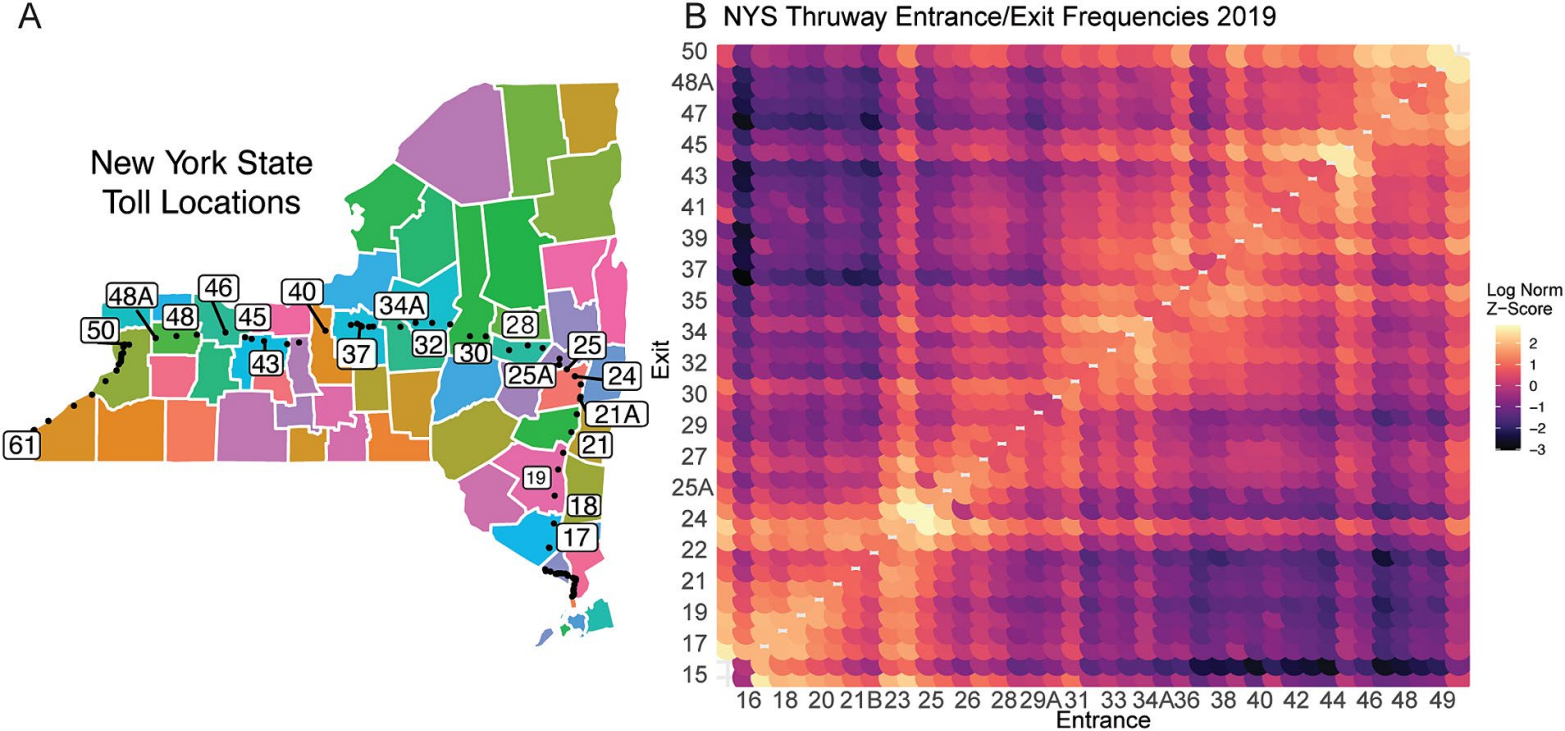
NYS broad travel patterns using traffic data. **(A)** Schematic of NYS Thruway Entrance and Exit points. Colors are used to denote unique county borders. Black dots represent each toll booth. Numbered exits are subset for visual clarity and regional significance. **(B)** Log Normalized entrance and exit contact matrix across NYS Thruway. Higher values indicate increased frequency of travel.

We next examined regional travel patterns, as population movement plays a pivotal role in the spread of infectious diseases. To establish a general model for large-scale population travel patterns, we leveraged NYS Thruway vehicle traffic data as a proxy to quantify travel behavior, which may connect distinct regions across NYS (Authority, N. Y. S. T, 2020). The main-line NYS Thruway spans 426 miles and runs from the WNY region (exit 50) to NYC (exit 15) with extensions into the North Country EDR (Figure 5A). We detect distinct commuter corridors linking different EDRs (Figure 5B). For example, the Capital District Region (Exits 23–26) shows an increased frequency of travel with the Mid-Hudson (Exits 16–21). (Figure 5B). Alternatively, exits 45–47 are a hub between the rest of the Western New York region (Exits 48–50) and the rest of the Finger Lakes and Central New York (Figure 5B). Furthermore, specific entrance points, like exit 50, show increased travel that spans the length of the thruway, representing travelers traversing the full extent of the NYS Thruway (Figure 5B). Although these data represent pre-pandemic travel from 2019, we predicted that the travel trends would have remained the same after the onset of COVID-19, even with overall reductions in travel. Notably, these commuter corridors were consistent with our lineage correlation analysis in 2020, where specific regions showed increased correlations in lineages circulating early in the pandemic (Figure 2B).

### 3.4 Agent-based disease SEIR modelling

**Figure 6:**
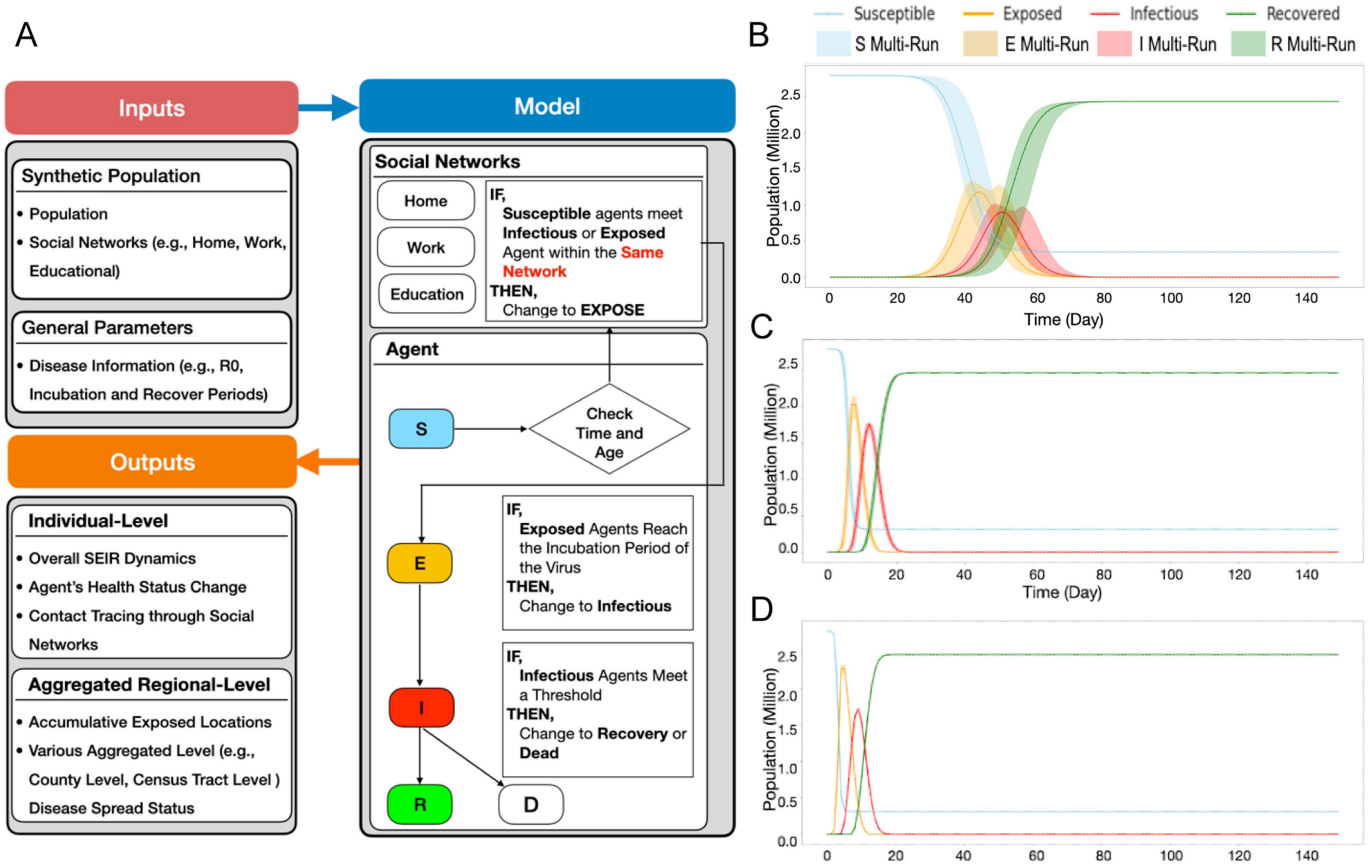
SEIR model schematic and dynamics. **(A)** Schematics of SEIR model including general parameter and synthetic population parameter sets, and model initialization and function **(B)** R0 = 3 Susceptibility, Exposed, Infectious, and Recovered curves based on the introduction of two infected agents, monitored over time. **(C)** R0 = 5, **(D)** R0 = 8.

**Figure 7:**
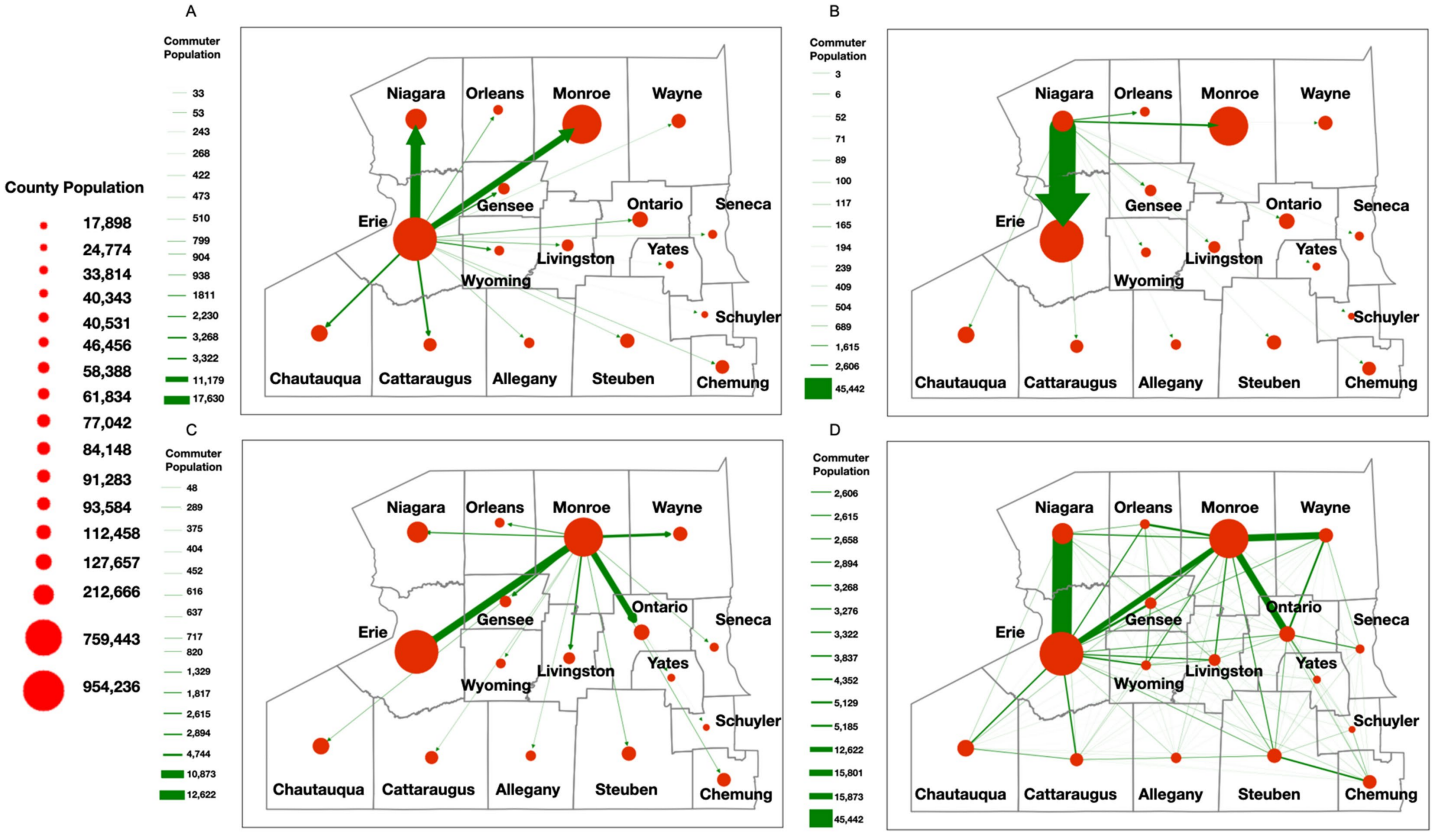
Commuter behavior dynamics in WNY. Estimated commuter populations originating in a specific county. **(A)** Commuter behavior with Erie County origins. **(B)** Commuter behavior from Niagara County origin. **(C)** Commuter behavior from Monroe County origin. **(D)** Composite Commuter behavior network.

While the NYS Thruway data helped establish broad regional travel dynamics, we sought to create a more finely tuned model of population-level movement dynamics within WNY. To accomplish this task, utilizing an agent-based SEIR model (described in more detail in the methods section), we simulated how a single SARS-CoV-2 lineage spreads through space and time based on individuals’ (i.e., agents’) social networks in the WNY Area (Figure 6A; Supplementary Figure 7). The purpose of the model is to demonstrate how commuter activity could lead to the diffusion of SARS-CoV-2 in WNY. To test our hypothesis that commuter activity promotes the pattern of diffusion of SARS-CoV-2 in WNY, we evaluated our SEIR model. We simulated the spread of a lineage for 50 days (i.e., 150-time steps) in the Western New York Area. To start the simulation, two agents from Erie County were selected as infected, and then the model was run. Figure 6B shows the overall SEIR dynamics based on the average results from 10 runs of the agent-based model, specifically, the line plot illustrates the average SEIR dynamics, while the shaded area represents the variances among the multiple runs. As expected SEIR dynamics is greatly influenced based on different R0 parameters (Figures 6C,D). To understand how commuting impacts the spread of the disease, we first analyzed the commuting patterns in WNY. If we focus on Erie County first, neighboring counties (Niagara and Monroe) have the bulk of intra-county commuters (Figure 7A). Niagara County residents mainly commuted to Erie County (Figure 7B), while Monroe County served as a major hub for commuters to several different regions, including Erie County, Ontario County, Wayne County, and Niagara County (Figure 7C). The overall inter-connected regional commutes are summarized in Figure 7D. These data suggest that regional transfer of SARS-CoV-2 lineages is likely in the Western New York region due to daily commuter activity connecting these communities.

**Figure 8:**
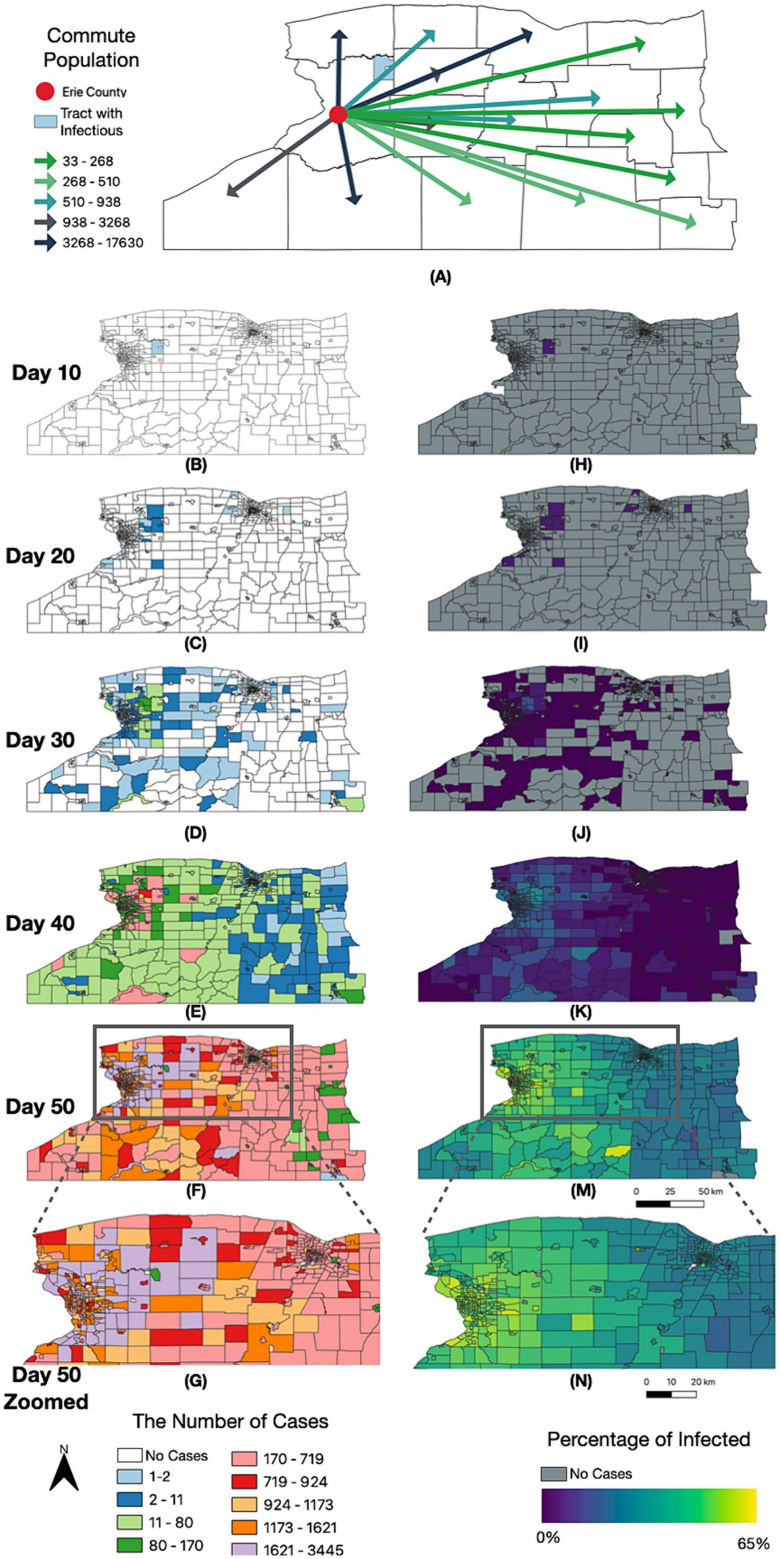
Spatially resolved SEIR modelling of WNY. Timelapse of infection case number and by percentage over 50 days, organized by NYS census tract. **(A)** Erie Commuting Population to Other Counties in NYS. **(B–G)** Number of total cases since D0 introduction. **(H–N)** Percentage of the census block having been infected with COVID-19 since D0 introduction.

For the SEIR model, we aggregated the results at the census tract level and conducted a set of spatial–temporal analyses to demonstrate the diffusion of SARS-CoV-2 led by commuters in WNY (Figure 8). Within 20 days post-introduction within Erie County, our model results reveal that several census tracts over 50 kilometers away in Monroe County saw signs of infection (Figures 8C,I). After 30 and 40 days, there was widespread infection across most counties in the Western New York, Southern Tier, and Finger Lakes regions, with a gradient of diffusion around the original infected census tract (Figures 8D–K). Finally, by day 50, our model suggested that all regions in WNY would harbor cases of the lineage introduced into our model, with an increase in cases forming a corridor between Erie County and Monroe County (Figures 8F,M,G,N). These results are consistent with our hypothesis that there is strong regional interconnectedness that would facilitate spread from Erie County and Monroe County metropolitan regions over a relatively brief period, and this spread is likely driven largely by commuter dynamics. We note that the simulation results are similar to the real-life diffusion of BA.2.12.1 across WNY in 2022 (Figure 4). In addition, we visually compared the model-generated disease spread map to the distribution of BA.1.1 (Omicron) cases in Western New York (Supplementary Figure 8). We chose a single lineage to visualize, representing a single R0 value, that originated in Erie County, as in our simulation. BA.1.1 spread through the WNY area during early 2022, a period of easing restrictions and return to normal activities, making it consistent with our simulation model. BA.1.1 spread quickly from Erie County into Niagara County, consistent with commuter patterns, and was detected in Genesee County within a few weeks. Our model captured a similar spread pattern, where the simulated results are in qualitative agreement with empirically derived disease distribution, which aligns with our current purpose as a proof of concept (Axtell and Epstein, 1994).

## 4 Discussion

### 4.1 Regional monitoring for understanding SARS-CoV-2 evolution

In this study, we established region-specific mutational patterns in patient sequencing data of SARS-CoV-2 at the sub-lineage level. Furthermore, we evaluated regional traffic and commuter patterns and their implications on the genetic diversity and spatial transmission of COVID-19 in New York State. Through location-aware agent-based modeling, we highlighted cross-county interactions that likely influence lineages circulating at the local level. It is our hope that these analyses will contribute to policymakers’ decisions during future outbreaks and support the benefits of local and regional monitoring of patient-level viral genomes.

New York State is made up of several dense urban metropolitan centers like New York City, the Capital Region, Utica, Syracuse, Rochester, and Buffalo, with nearly 20 million people as of 2022 (U.S Census Bureau, 2022). In between these dense urban centers are rural communities that our analysis reveals serve as commuting hubs for neighboring counties. Focusing our analysis on Western New York, our SEIR model suggests the ability of SARS-CoV-2 to diffuse across these rural communities, leading to the transmission from one metropolitan region to another (e.g., Rochester, NY to Buffalo, NY). While our model did not incorporate vaccination rates, it is worth noting that rural counties between Erie and Monroe tend to have lower percentages of people who have finished the complete course of vaccinations and also tend to be less likely to have the updated booster formulations (Health, N. Y. S. D. O, 2023). At the same time, there is a dearth of genomic sequencing in these regions (Supplementary Figures 2–4, 9). Therefore, our models can help predict diffusion patterns of infectious agents into these areas of the state.

As with all models, there are several limitations and thus areas of further work. First, the synthetic population only captures basic patterns of life, in the sense our agents only go to work, educational sites or home, where they have the potential to be exposed or infected. Future work could explore other types of activities such as meeting friends, going shopping, etc. at a finer temporal scale. This could be informed by data from other sources (e.g., SafeGraph or the National Household Travel Survey). However, to achieve this, it requires altering the agent behavior based on what motivates people to carry out different activities, which is beyond the scope of this work. Furthermore, our model did not incorporate detailed demographic information, such as people aged over 65 who are retired and stay at home but remain vulnerable to the SARS-CoV-2. Such information could be extracted from the American Community Survey. We do note that ~60% of seniors are in the workforce. Furthermore, those agents who are retired are connected to others via the various social networks we use in the model. Another area of further work for our model is to quantitatively assess disease dynamics that the model produces with actual COVID-19 infection rates from the Centers for Disease Control and Prevention (CDC), to tune the model to mimic the effects of lockdowns and other interventions to inform planning scenarios for future pandemics. These limitations notwithstanding, the model presented in this paper captures basic disease dynamics and helps predict diffusion patterns of infectious agents in specific geographic locations, which could provide the foundation for researchers to explore the next pandemic utilizing agent-based modeling and synthetic population with social networks.

Our genetic analysis of SARS-CoV-2 samples across NYS uncovered regional differences in the genetic backgrounds within specific lineages. We note that lineage profiles became more homogeneous as masking, distancing, and travel restriction policies were relaxed through 2021 and especially 2022 (Figure 2B; Supplementary Figure 5). Together, these observations indicate the importance and utility of targeted, regional public health policies. Furthermore, while significant resources were invested into regional sequencing hubs, a disproportionate amount of sequencing data was generated downstate in the New York City compared to more rural counties, like the Southern Tier and the North Country (Supplementary Figure 9). Our analysis demonstrates that even within a single SARS-CoV-2 lineage (like B.1.1.7), distinct genetic diversity exists that could lead to changes in transmission rates at the local level. Due to this, continued investment in the infrastructure needed for sample acquisition and monitoring in rural communities outside of dense urban centers is critical for the ongoing COVID-19 pandemic and future outbreaks of both novel and known infectious diseases.

Despite the prevalence of SARS-CoV-2 infections, sample collection and sequencing of patient-derived samples have decreased since the height of the pandemic. The widespread availability of at-home diagnostic tools has reduced collection rates (Rader et al., 2022). Furthermore, many pandemic monitoring groups have adopted wastewater-based approaches (Karthikeyan et al., 2022; Segelhurst et al., 2023; Wilder et al., 2021). While wastewater serves as a viable tool for measuring overall infection rates, and deconvolution techniques indicate relative proportions of lineages, broad monitoring through wastewater introduces a gap in data for localities without municipal treatment facilities, although, recent analysis suggests the feasibility of the method for rural communities (Conway et al., 2023). Alternative methods such as SEIR modeling used in this study could serve as potential surrogate strategies. Indeed, recent surveys of residents of New York State indicate that over 70% of SARS-CoV-2 infections are diagnosed exclusively by at-home tests, further reinforcing the benefit of computational models such as ours (Mitchell et al., 2023).

In conclusion, our study sheds light on the intricate dynamics of the COVID-19 pandemic within the Western region of New York State, emphasizing the importance of understanding local transmission dynamics alongside the broader global perspective. Combining spatially informed SEIR models and detailed genomic analysis of SARS-CoV-2 lineages provides a comprehensive approach to understanding regional transmission networks. Our analysis of statewide SARS-CoV-2 lineages over time reveals distinct regional differences, especially early in the pandemic. Furthermore, our investigation of single nucleotide polymorphisms within specific VOC lineages exposed localized genomic alterations typically obscured by aggregation into broad lineage designations, which can be leveraged for genomic epidemiology to monitor and understand infection spread. Our findings thus underscore the benefits of regional monitoring, genetic diversity analysis, and spatial modeling. As the pandemic continues to evolve, we hope this integrative analysis offers valuable insights for policymakers and health officials to implement targeted interventions and allocate resources efficiently and effectively.

## Data Availability

The datasets presented in this study can be found in online repositories. The names of the repository/repositories and accession number(s) can be found in the article/Supplementary material.

## References

[ref1] Achaiah N. C. Subbarajasetty S. B. Shetty R. M. (2020). R(0) and R(e) of Covid-19: can we predict when the pandemic outbreak will be contained? Indian J. Crit. Care Med. 24, 1125–1127. doi: 10.5005/jp-journals-10071-23649, PMID: 33384521 PMC7751056

[ref2] Alpert T. Brito A. F. Lasek-Nesselquist E. Rothman J. Valesano A. L. Mackay M. J.. (2021). Early introductions and transmission of Sars-CoV-2 variant B.1.1.7 in the United States. Cell 184, 2595–2604.e13. doi: 10.1016/j.cell.2021.03.061, PMID: 33891875 PMC8018830

[ref3] Authority, B. A. F. E. P. B (2023). Yearly volumes [online]. Buffalo and Fort Erie Public Bridge Authority. Available at: https://www.peacebridge.com/index.php/historical-traffic-statistics/yearly-volumes (Accessed March 12, 2023).

[ref4] Authority, N. Y. S. T (2020). Nys thruway origin and destination points for all vehicles −1 hour intervals: 2019 [online]. New York state thruway Authority. Available at: https://data.ny.gov/Transportation/Nys-Thruway-Origin-and-Destination-Points-for-All-/chzq-388p (Accessed December 19, 2022).

[ref5] Axtell R. L. Epstein J. (1994). Agent-based modeling: understanding our creations.

[ref6] Beheshti Namdar A. Keikha M. (2022). Ba.2.12.1 is a new omicron offshoot that is a highly contagious but not severe disease. Ann. Med. Surg. 79:104034. doi: 10.1016/j.amsu.2022.104034, PMID: 35770273 PMC9234247

[ref7] Bogner P. Capua I. Lipman D. J. Cox N. J. (2006). A global initiative on sharing avian flu data. Nature 442:981. doi: 10.1038/442981a

[ref8] Cao Y. Yisimayi A. Jian F. Song W. Xiao T. Wang L.. (2022). Ba.2.12.1, Ba.4 and Ba.5 escape antibodies elicited by omicron infection. Nature 608, 593–602. doi: 10.1038/s41586-022-04980-y, PMID: 35714668 PMC9385493

[ref9] Conway M. J. Kado S. Kooienga B. K. Sarette J. S. Kirby M. H. Marten A. D.. (2023). Sars-CoV-2 wastewater monitoring in rural and small metropolitan communities in Central Michigan. Sci. Total Environ. 894:165013. doi: 10.1016/j.scitotenv.2023.165013, PMID: 37353028

[ref10] Crooks A. Malleson N. Manley E. Heppenstall A. J. (2019). Agent-based modelling & geographical information systems: a practical primer. Los Angeles: SAGE Publications.

[ref11] Dunbar R. I. M. (1998). The social brain hypothesis. Evol. Anthropol. Issues News Rev. 6, 178–190. doi: 10.1002/(SICI)1520-6505(1998)6:5<178::AID-EVAN5>3.0.CO;2-8

[ref12] Emanuel E. J. Persad G. Upshur R. Thome B. Parker M. Glickman A.. (2020). Fair allocation of scarce medical resources in the time of Covid-19. N. Engl. J. Med. 382, 2049–2055. doi: 10.1056/NEJMsb2005114, PMID: 32202722

[ref13] Foraker R. E. Lai A. M. Kannampallil T. G. Woeltje K. F. Trolard A. M. Payne P. R. O. (2021). Transmission dynamics: data sharing in the Covid-19 era. Learn. Health Syst. 5:e10235. doi: 10.1002/lrh2.10235, PMID: 32838037 PMC7323052

[ref14] Grolemund G. Wickham H. (2011). Dates and times made easy with lubridate. J. Stat. Softw. 40, 1–25. doi: 10.18637/jss.v040.i03

[ref15] Health, N. Y. S. D. O (2023). Zip Code Vaccination Data [Online]. New York State Department of Health. Available at: https://coronavirus.health.ny.gov/zip-code-vaccination-data (Accessed March 12, 2023).

[ref16] Jiang N. Crooks A. T. Kavak H. Burger A. Kennedy W. G. (2022). A method to create a synthetic population with social networks for geographically-explicit agent-based models. Comput. Urban Sci. 2:7. doi: 10.1007/s43762-022-00034-1

[ref17] Karthikeyan S. Levy J. I. De Hoff P. Humphrey G. Birmingham A. Jepsen K.. (2022). Wastewater sequencing reveals early cryptic Sars-CoV-2 variant transmission. Nature 609, 101–108. doi: 10.1038/s41586-022-05049-6, PMID: 35798029 PMC9433318

[ref18] Katoh K. Rozewicki J. Yamada K. D. (2019). Mafft online service: multiple sequence alignment, interactive sequence choice and visualization. Brief. Bioinform. 20, 1160–1166. doi: 10.1093/bib/bbx108, PMID: 28968734 PMC6781576

[ref19] Khare S. Gurry C. Freitas L. Schultz M. B. Bach G. Diallo A.. (2021). Gisaid’s role in pandemic response. China CDC Wkly. 3, 1049–1051. doi: 10.46234/ccdcw2021.255, PMID: 34934514 PMC8668406

[ref20] Kong L. Duan M. Shi J. Hong J. Chang Z. Zhang Z. (2022). Compartmental structures used in modeling Covid-19: a scoping review. Infect. Dis. Poverty 11:72. doi: 10.1186/s40249-022-01001-y, PMID: 35729655 PMC9209832

[ref21] Mitchell E. C. Nguyen T. Boulais M. Ravi Brenner I. Dorabawila V. Hoen R.. (2023). Home testing for Sars-CoV-2 and impact on surveillance in New York State. Ann. Epidemiol. 91, 74–81. doi: 10.1016/j.annepidem.2023.11.009, PMID: 37995986

[ref22] Miyah Y. Benjelloun M. Lairini S. Lahrichi A. (2022). Covid-19 impact on public health, environment, human psychology, global socioeconomy, and education. Sci. World J. 2022:5578284. doi: 10.1155/2022/5578284, PMID: 35069037 PMC8767375

[ref23] Newman M. E. J. Watts D. J. (1999). Renormalization group analysis of the small-world network model. Phys. Lett. A 263, 341–346. doi: 10.1016/S0375-9601(99)00757-4

[ref24] Price M. N. Dehal P. S. Arkin A. P. (2010). FastTree 2 – approximately maximum-likelihood trees for large alignments. PLoS One 5:e9490. doi: 10.1371/journal.pone.0009490, PMID: 20224823 PMC2835736

[ref25] Quinlan A. R. Hall I. M. (2010). Bedtools: a flexible suite of utilities for comparing genomic features. Bioinformatics 26, 841–842. doi: 10.1093/bioinformatics/btq033, PMID: 20110278 PMC2832824

[ref26] Rader B. Gertz A. Iuliano A. D. Gilmer M. Wronski L. Astley C. M.. (2022). Use of at-home covid-19 tests—United States, august 23, 2021–March 12, 2022. Morb. Mortal. Wkly Rep. 71, 489–494. doi: 10.15585/mmwr.mm7113e1, PMID: 35358168 PMC8979595

[ref27] Rambaut A. Holmes E. C. O’toole Á. Hill V. Mccrone J. T. Ruis C.. (2020). A dynamic nomenclature proposal for Sars-CoV-2 lineages to assist genomic epidemiology. Nat. Microbiol. 5, 1403–1407. doi: 10.1038/s41564-020-0770-5, PMID: 32669681 PMC7610519

[ref28] Sawicka B. Aslan I. Della Corte V. Periasamy A. Krishnamurthy S. K. Mohammed A.. (2022). “Chapter 14- the coronavirus global pandemic and its impacts on society” in Coronavirus drug discovery. ed. Egbuna C. (Amsterdam: Elsevier).

[ref29] Segelhurst E. Bard J. E. Pillsbury A. N. Alam M. M. Lamb N. A. Zhu C.. (2023). Robust performance of Sars-CoV-2 whole-genome sequencing from wastewater with a nonselective virus concentration method. ACS ES T Water 3, 954–962. doi: 10.1021/acsestwater.2c00456, PMID: 37406038 PMC10005814

[ref30] Shankar S. Mohakuda S. S. Kumar A. Nazneen P. S. Yadav A. K. Chatterjee K.. (2021). Systematic review of predictive mathematical models of Covid-19 epidemic. Med. J. Armed Forces India 77, S385–s392. doi: 10.1016/j.mjafi.2021.05.005, PMID: 34334908 PMC8313025

[ref31] Tamarit I. Sánchez A. Cuesta J. A. (2022). Beyond Dunbar circles: a continuous description of social relationships and resource allocation. Sci. Rep. 12:2287. doi: 10.1038/s41598-022-06066-1, PMID: 35145151 PMC8831677

[ref32] U.S Census Bureau. (2022). QuickFacts New York [online]. United States Census Bureau. Available at: https://www.census.gov/quickfacts/fact/table/Ny/Pst045222 (Accessed March 12, 2023).

[ref33] Vallée A. (2023). Geoepidemiological perspective on Covid-19 pandemic review, an insight into the global impact. Front. Public Health 11:1242891. doi: 10.3389/fpubh.2023.1242891, PMID: 37927887 PMC10620809

[ref34] Wang L. G. Lam T. T. Xu S. Dai Z. Zhou L. Feng T.. (2020). Treeio: an R package for phylogenetic tree input and output with richly annotated and associated data. Mol. Biol. Evol. 37, 599–603. doi: 10.1093/molbev/msz240, PMID: 31633786 PMC6993851

[ref35] West B. J. Massari G. F. Culbreth G. Failla R. Bologna M. Dunbar R. I. M.. (2020). Relating size and functionality in human social networks through complexity. Proc. Natl. Acad. Sci. USA 117, 18355–18358. doi: 10.1073/pnas.2006875117, PMID: 32690712 PMC7414177

[ref36] Wickham H. (2016). Ggplot2: Elegant graphics for data analysis. Cham, Switzerland: Springer International Publishing.

[ref37] Wilder M. L. Middleton F. Larsen D. A. Du Q. Fenty A. Zeng T.. (2021). Co-quantification of crAssphage increases confidence in wastewater-based epidemiology for Sars-CoV-2 in low prevalence areas. Water Res. X 11:100100. doi: 10.1016/j.wroa.2021.100100, PMID: 33842875 PMC8021452

[ref38] Wong H. S. Hasan M. Z. Sharif O. Rahman A. (2023). Effect of total population, population density and weighted population density on the spread of Covid-19 in Malaysia. PLoS One 18:e0284157. doi: 10.1371/journal.pone.0284157, PMID: 37104371 PMC10138265

[ref39] Wu Y. Kang L. Guo Z. Liu J. Liu M. Liang W. (2022). Incubation period of Covid-19 caused by unique Sars-CoV-2 strains: a systematic review and meta-analysis. JAMA Netw. Open 5:e2228008. doi: 10.1001/jamanetworkopen.2022.28008, PMID: 35994285 PMC9396366

[ref40] Wurmb T. Scholtes K. Kolibay F. Schorscher N. Ertl G. Ernestus R. I.. (2020). Hospital preparedness for mass critical care during Sars-CoV-2 pandemic. Crit. Care 24:386. doi: 10.1186/s13054-020-03104-0, PMID: 32605581 PMC7325193

[ref41] Yu G. (2020). Using ggtree to visualize data on tree-like structures. Curr. Protoc. Bioinformatics 69:e96. doi: 10.1002/cpbi.96, PMID: 32162851

