# Supplementary material for: Genomic profiling and spatial SEIR modeling of COVID-19 transmission in Western New York
Source: Front Microbiol. 2024 Sep 27;15:1416580. doi: 10.3389/fmicb.2024.1416580 (PMC11468862; doi:10.3389/fmicb.2024.1416580)
Supplement: Supplementary file 5 [file Data_Sheet_1.pdf]

Supplementary Material

Genomic Profiling and Spatial SEIR Modeling of COVID-19 Transmission in Western New York.

Jonathan E. Bard*[1,2], Na Jiang[3]*, Jamaal Emerson[4], Madeleine Bartz[2], Natalie A. Lamb[5], Brandon J. Marzullo[2], Alyssa Pohlman[2], Amanda Boccolucci[2], Norma J. Nowak[1,2], Donald A. Yergeau[2], Andrew T. Crooks[3], Jennifer A. Surtees[1.4#]

1 Department of Biochemistry, Jacobs School of Medicine and Biomedical Sciences, State University of New York at Buffalo, Buffalo, NY, United States

2 Genomics and Bioinformatics Core, Jacobs School of Medicine and Biomedical Sciences, State University of New York at Buffalo, Buffalo, NY, United States

3 Department of Geography, State University of New York at Buffalo, Buffalo, NY, United States

4 Department of Microbiology and Immunology, Jacobs School of Medicine and Biomedical Sciences, State University of New York at Buffalo, Buffalo, NY, United States

5 National Renewable Energy Laboratory, Golden Colorado, United States

[*] These authors contributed equally

[#] Correspondence:
Jennifer A. Surtees
jsurtees@buffalo.edu

1.1 Supplementary Figures

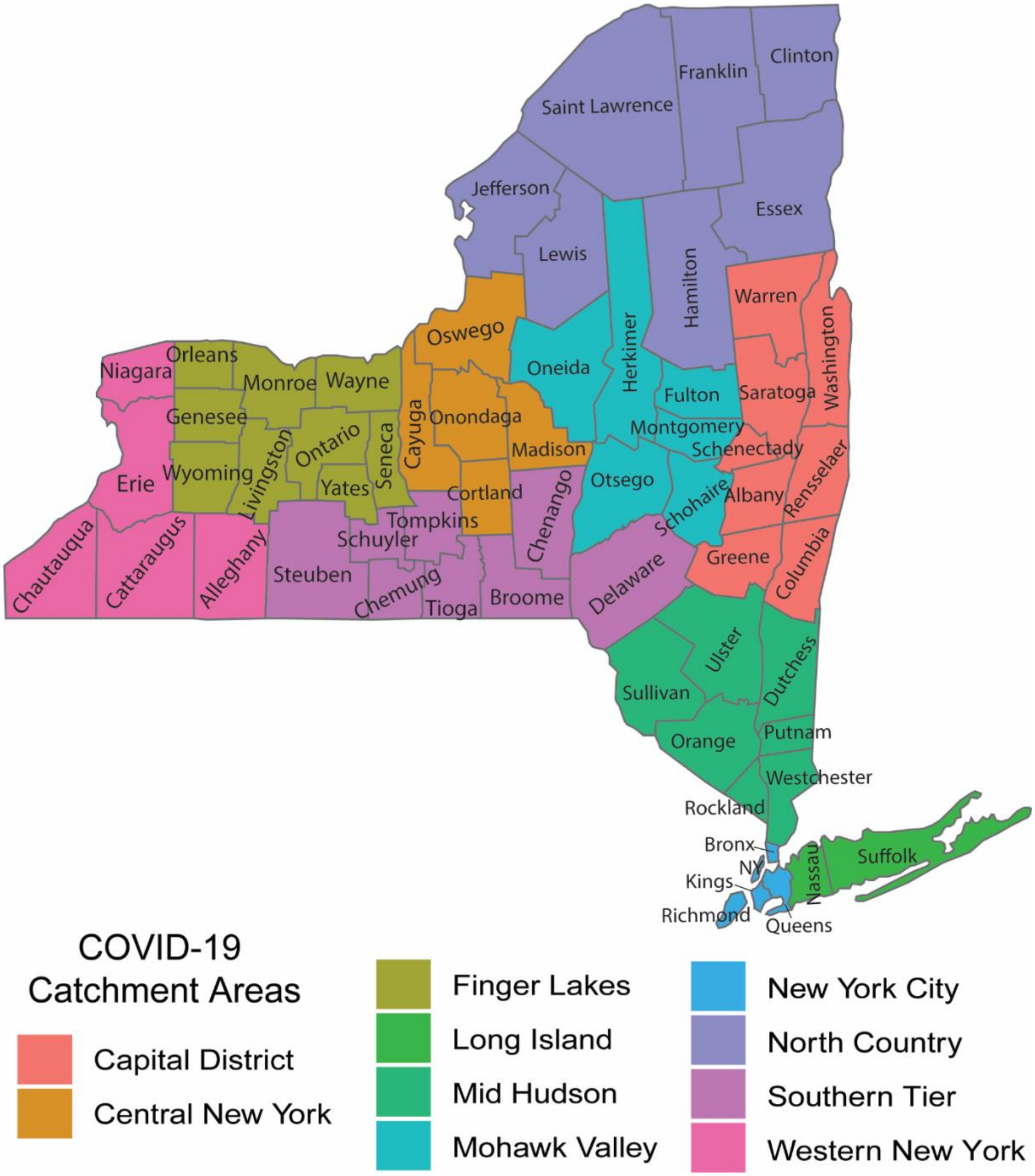

Supplemental Figure 1. NYS County Map and Economic Development Regions (EDRs). Spatial organization of the 68 counties of New York State, grouped by economic development region and COVID catchment groups.

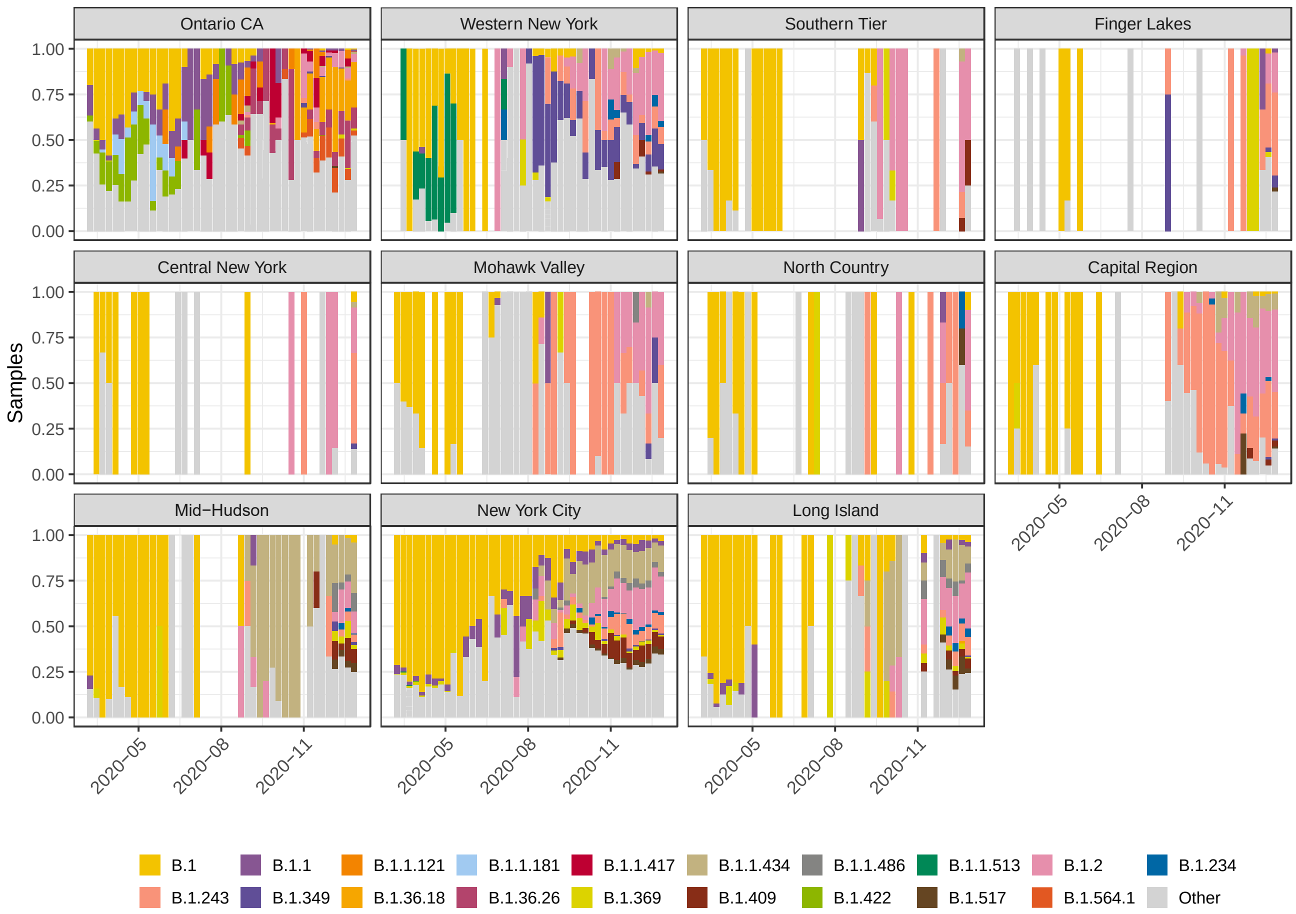

Supplemental Figure 2. Circulating Lineages in 2020. Lineage distributions by percentage of total cases within region per week across the 10 EDRs and Ontario Canada. The "Other" category represents the collection of lineages present with <1% of the total lineages and lacking discrete regional patterning.

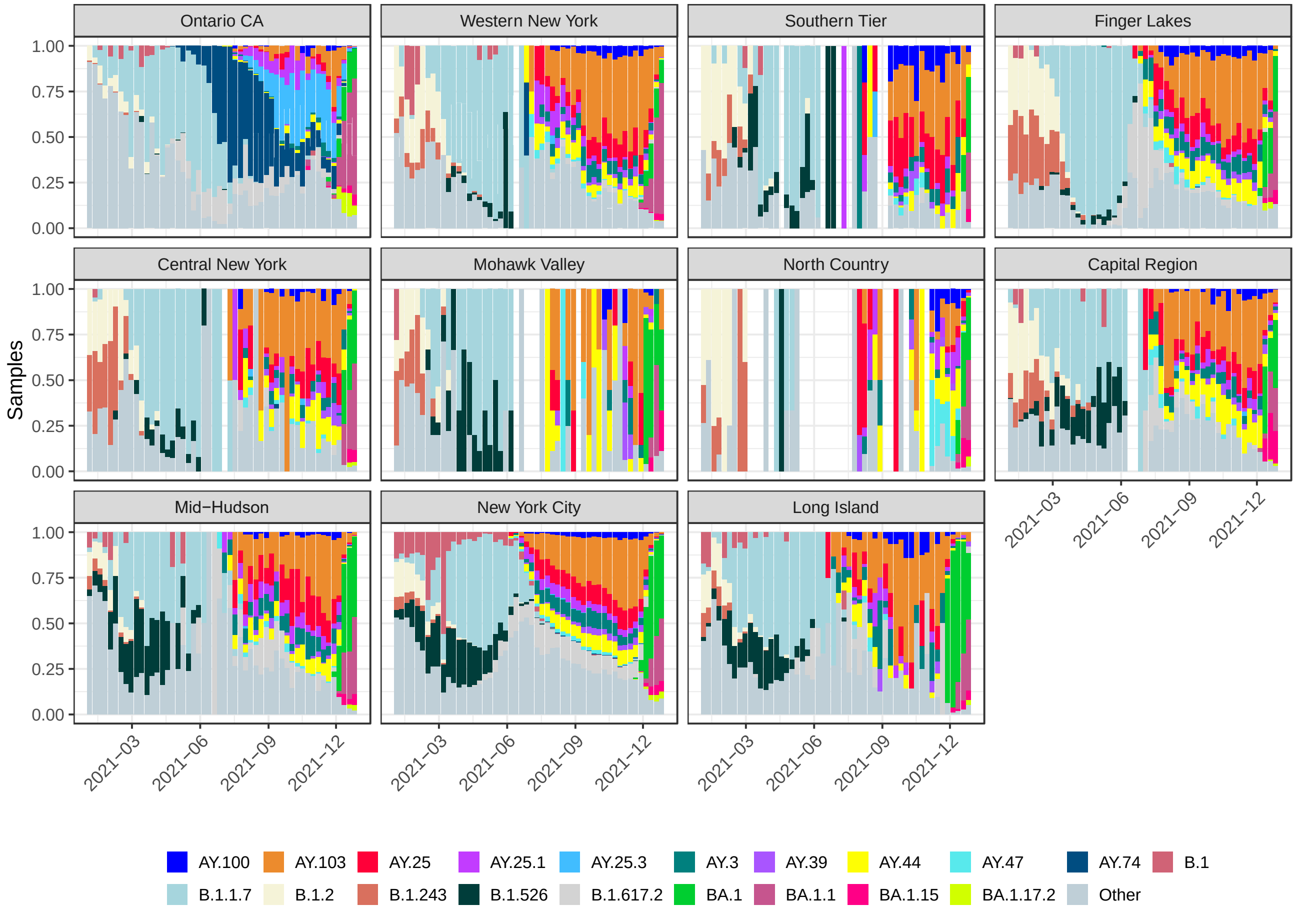

Supplemental Figure 3. Circulating Lineages in 2021. Lineage distributions by percentage of total cases within region per week across the 10 EDRs and Ontario Canada. The "Other" category represents the collection of lineages present with <1% of the total lineages and lacking discrete regional patterning.

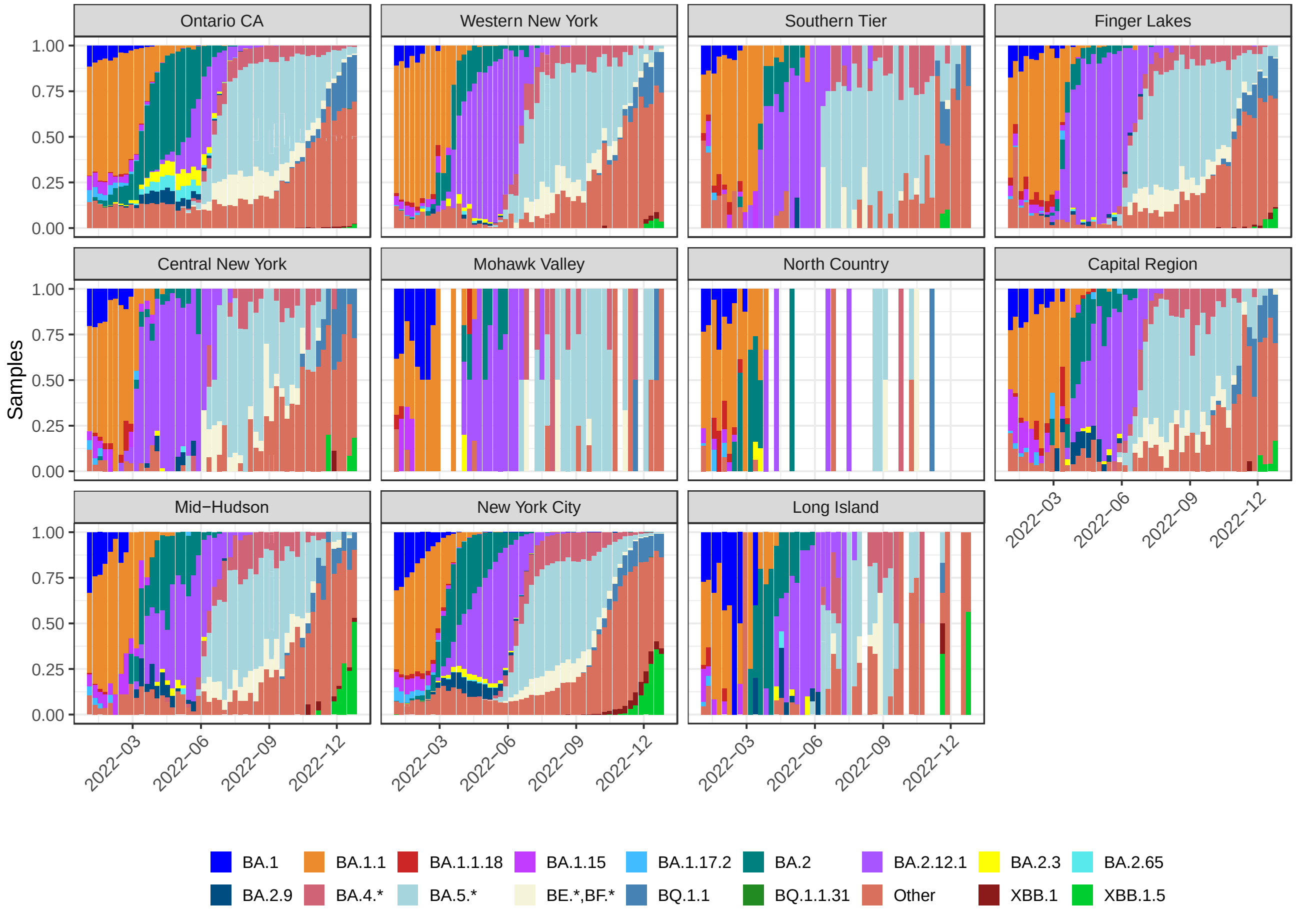

Supplemental Figure 4. Circulating Lineages in 2022. Lineage distributions by percentage of total cases within region per week across the 10 EDRs and Ontario Canada. The "Other" category represents the collection of lineages present with <1% of the total lineages and lacking discrete regional patterning.

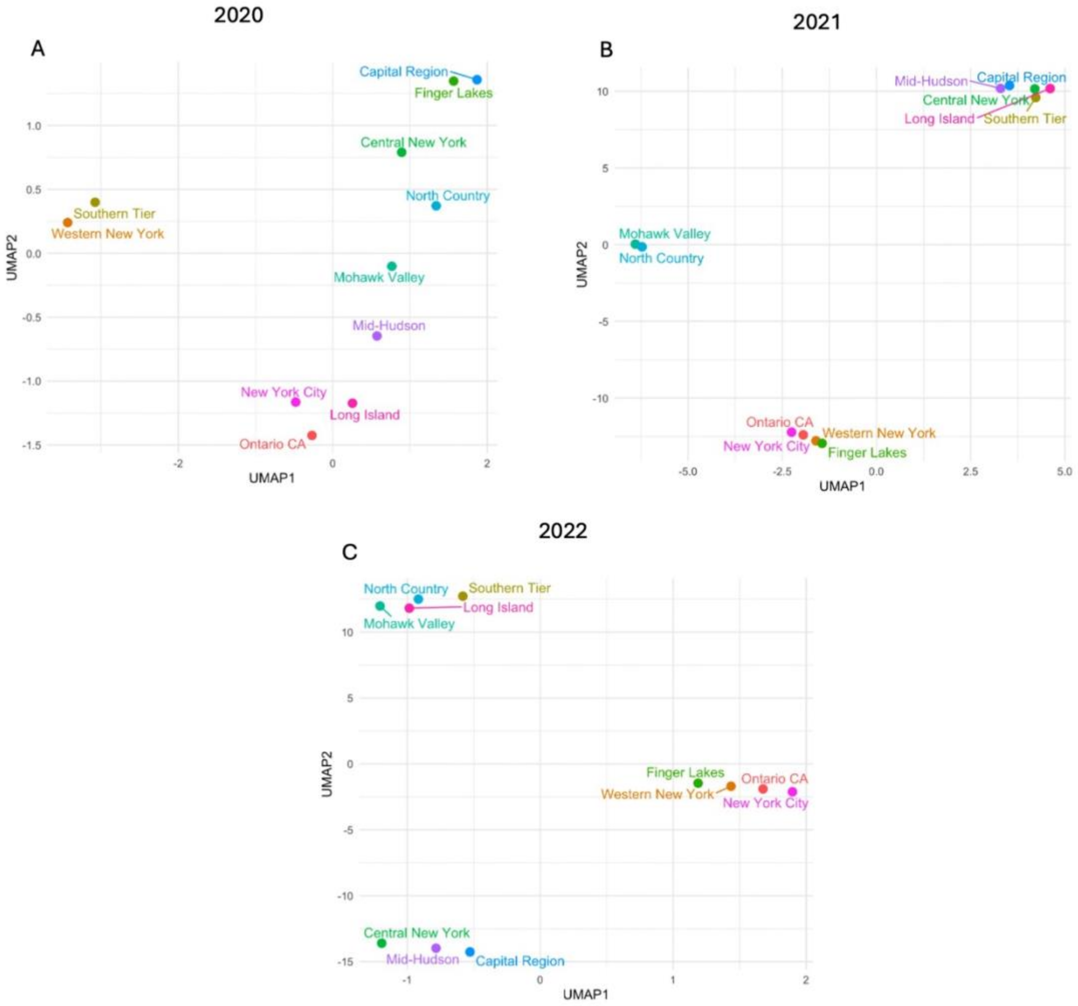

Supplemental Figure 5. Uniform Manifold Approximation and Projection (UMAP) reductions per EDR Region. A region-by-lineage matrix was generated with values corresponding to the yearly total of all samples assigned to that region.

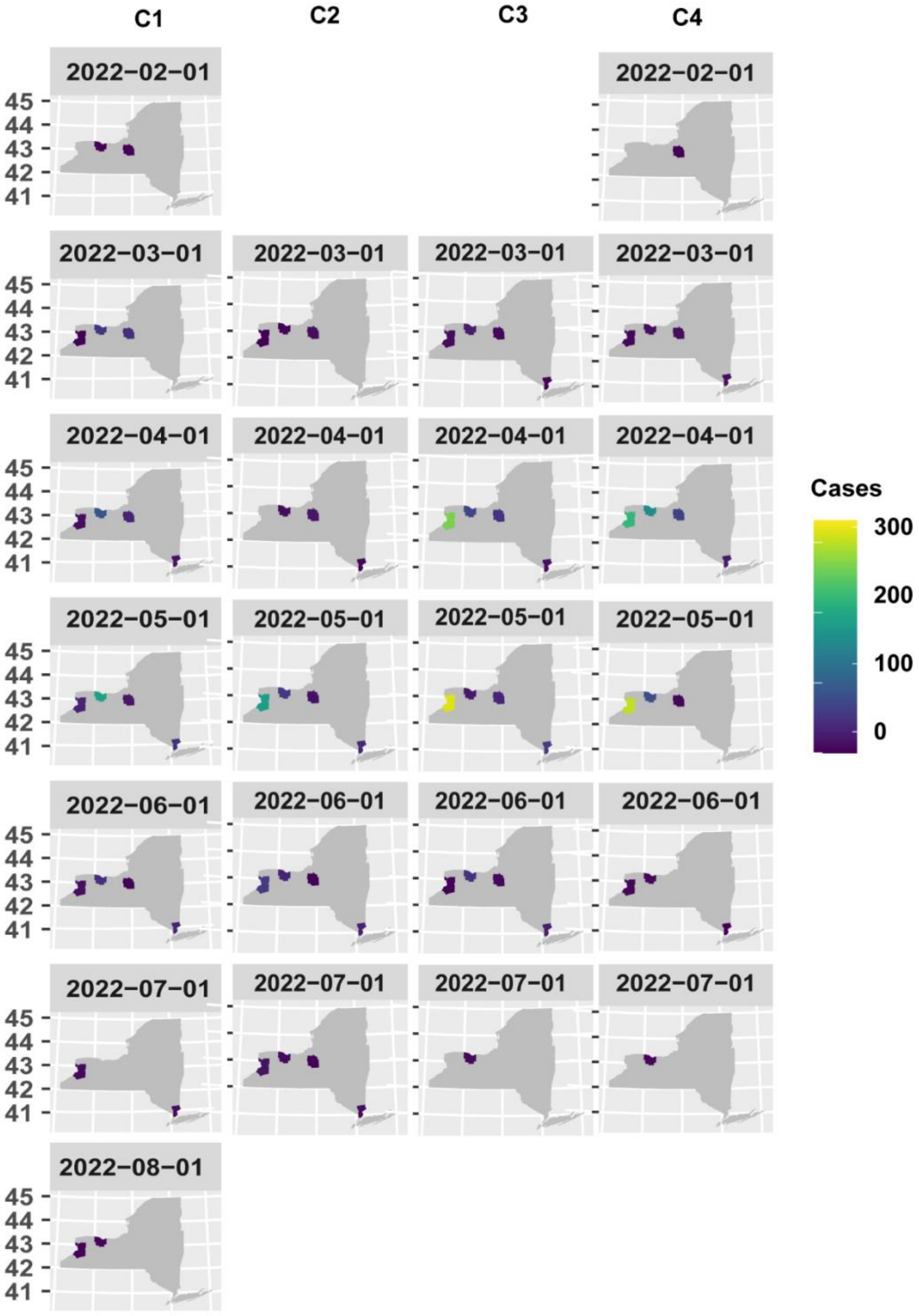

Supplemental Figure 6. BA.2.12.1 Spatial-temporal Modeling of K-Means Clustered Samples. Cluster 1-4 (C1,C2,C3,C4) reveal distinct temporal detection and subsequent extinction across Erie, Monroe, Onondaga, and Westchester Counties. Earliest time points are at the top of each column. Y-axis labels correspond to geographic latitude.

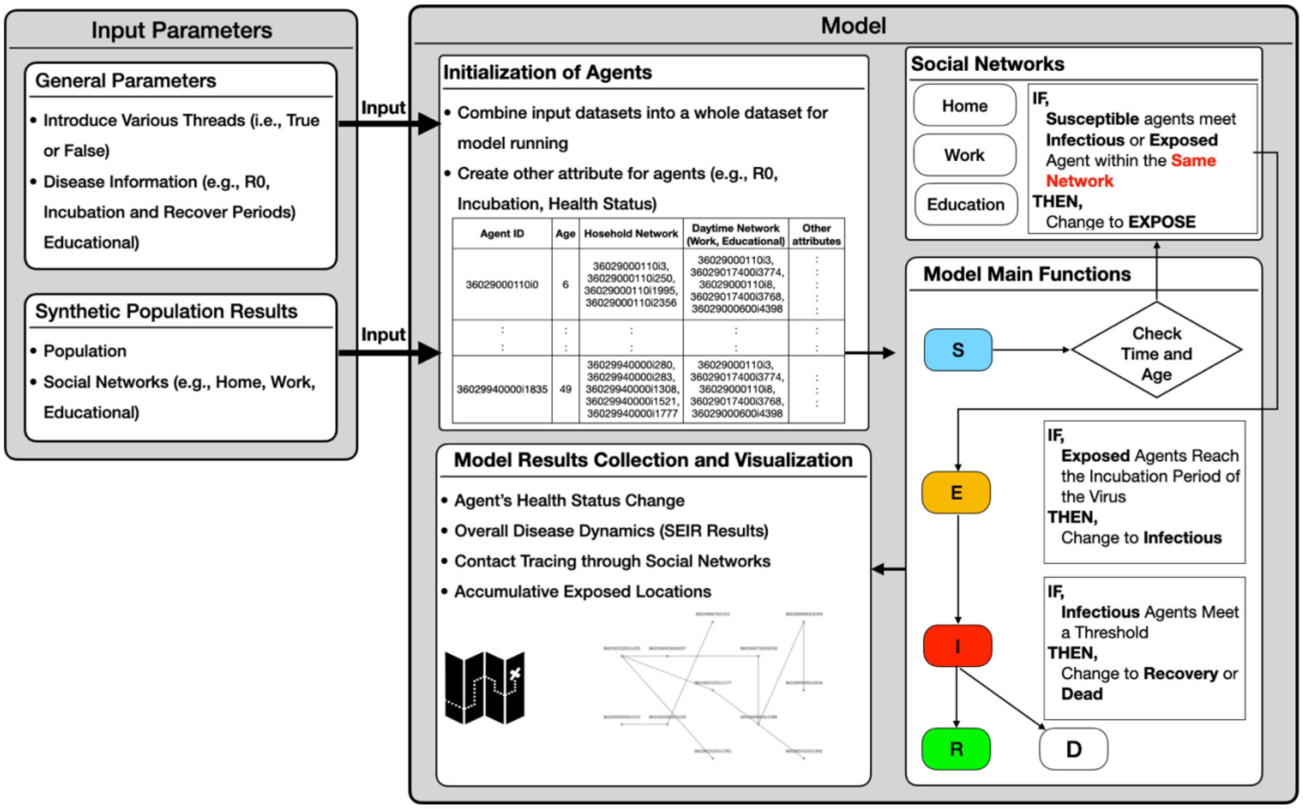

Supplemental Figure 7. SEIR Model Schematic. Detailed schematic of the SEIR model including general parameter and synthetic population parameter sets, as well as model initialization and function.

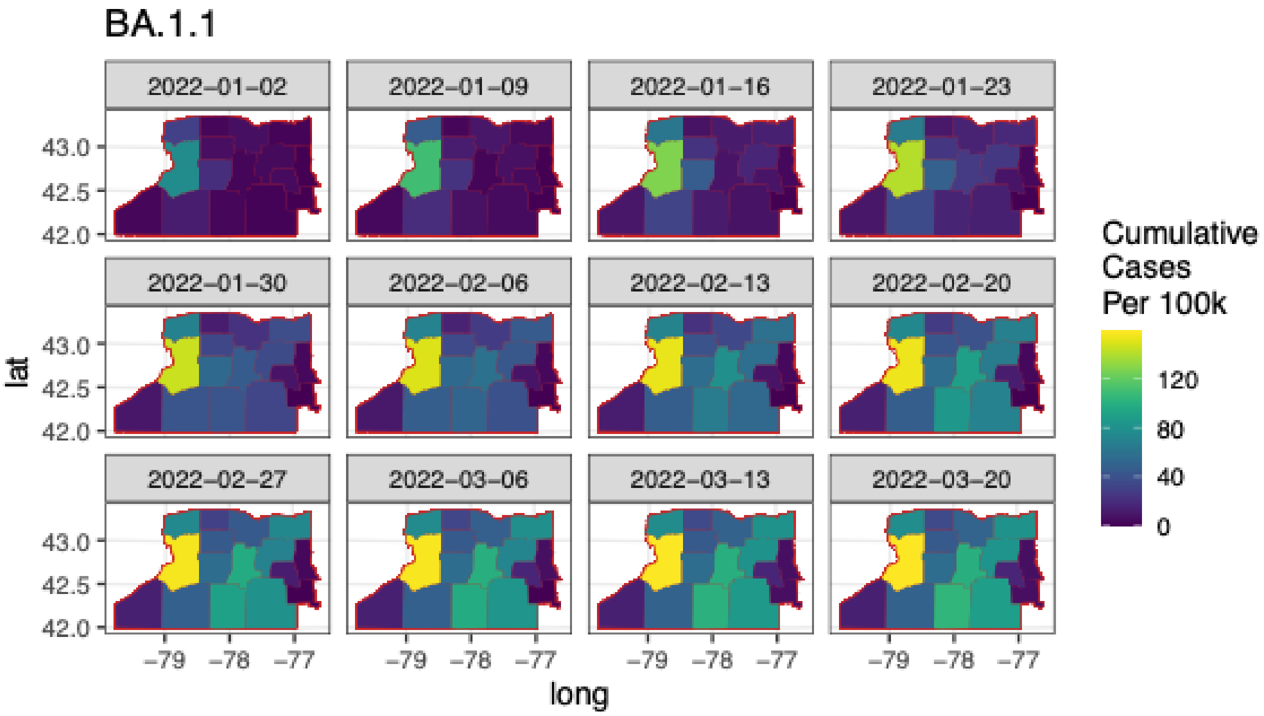

Supplemental Figure 8. BA.1.1 Weekly Transmission Patterns. January 2022 to March 2024 cumulative county total number of viral samples sequenced, normalized per 100,000 residents.

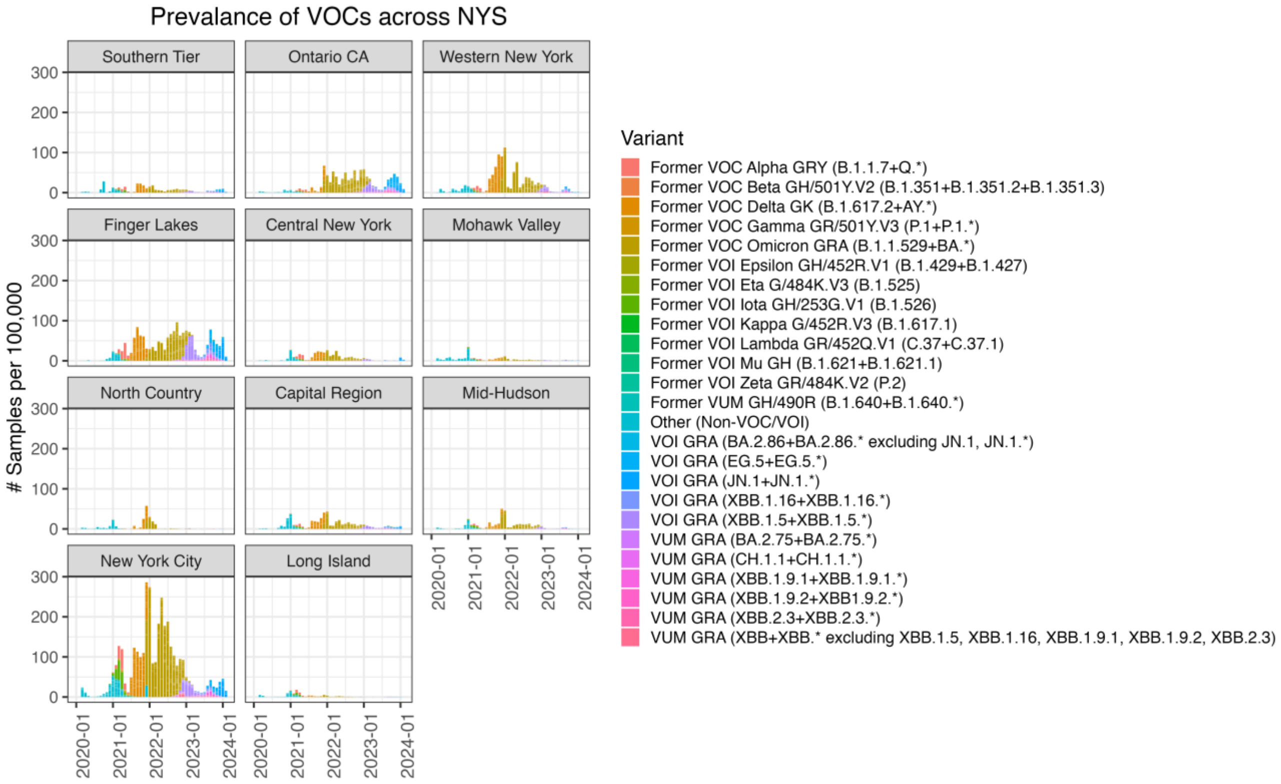

Supplemental Figure 9. Viral Genomes Sequenced by EDR Reveal Disproportionate Sequencing across NYS. January 2020 to January 2024 total number of viral samples sequenced, organized by EDR, normalized per 100,000 residents.
